# Pan-cancer analysis reveals immunological and prognostic significance of CCT5 in human tumors

**DOI:** 10.1038/s41598-025-88339-z

**Published:** 2025-04-24

**Authors:** Md. Zabir Ahmed, Md Mohtasim Billah, Jannatul Ferdous, Shoriful Islam Antar, Abdullah Al Mamun, Md. Jubayer Hossain

**Affiliations:** 1 Center for Health Innovation, Research, Action, and Learning—Bangladesh (CHIRAL Bangladesh), Dhaka, Bangladesh; 2 Big Bioinformatics Lab (BigBio Lab), Center for Health Innovation, Research, Action, and Learning— Bangladesh (CHIRAL Bangladesh), Dhaka, Bangladesh; 3 Department of Microbiology, Jagannath University, Dhaka, Bangladesh; 4 Department of Microbiology, Jahangirnagar University, Savar, Dhaka, Bangladesh; 5 Department of Animal Science and Veterinary Medicine, Bangabandhu Sheikh Mujibur Rahman Science and Technology University, Gopalganj, Bangladesh

**Keywords:** Cancer biology, Human tumors, CCT5, Biomarker, Pan-cancer analysis, Oncogenesis, Biotechnology, Cancer, Cell biology, Chemical biology, Computational biology and bioinformatics, Genetics, Immunology

## Abstract

The chaperonin containing TCP1 subunit 5 (CCT5) is believed to function as a tumor driver. However, a systematic pan-cancer analysis of CCT5 is still lacking. Therefore, this study aimed to identify the potential role of CCT5 in different types of tumors. This study comprehensively investigated the gene expression, proteomic expression, immune infiltration, DNA methylation, genetic alterations, correlation with TMB and MSI, drug sensitivity, enrichment analysis, and prognostic significance of CCT5 in 33 different tumors based on the TIMER2.0, GEPIA2, UALCAN, SMART, cBioPortal, GSCA databases, and *TCGAplot* R package. The results revealed significant CCT5 overexpression in most tumors and was significantly associated with poor OS and DFS in different tumor types. Reduced promoter and N-shore methylation of CCT5, indicating its potential oncogenic and epigenetic roles. Amplification was the most common type of CCT5 alterations. Immune infiltration analysis revealed a strong correlation between CCT5 and different immune cells. CCT5 exhibited a significant correlation with TMB and MSI in KIRC and STAD. Furthermore, enrichment analysis revealed associations between CCT5 and cell cycle pathway and various cellular functions. These findings suggested that CCT5 might serve as a potential prognostic biomarker and target for immunotherapy in various cancers.

**Supplementary Information:**

The online version contains supplementary material available at 10.1038/s41598-025-88339-z.

## Introduction

Cancer is a significant global health concern and is the second leading cause of death worldwide^1^. In 2022 alone, an estimated 20 million new cases were diagnosed, and tragically, 9.7 million individuals died of the disease. Nearly half of all new cancer cases have been reported in Asia^2^. Consequently, it is crucial to investigate the molecular mechanisms of tumorigenesis and to identify potential biomarkers for cancer diagnosis and prognosis. By leveraging advanced techniques and numerous public resources, cancer bioinformatics enables us to conduct comprehensive pan-cancer analyses to explore cancer-associated genes of interest.

Chaperonin-containing tailless complex polypeptide 1 (CCT), also known as the TCP1 ring complex (TRiC), is a protein complex that plays a well-established role in cancer development via folding of the cytoskeletal protein actin and tubulin^3–6^. The CCT family consists of nine subunits responsible for facilitating protein folding, either individually or in oligomeric complexes^3,5,7^.

CCT5 encodes a subunit of the TRiC complex, and its ability to translate into a functional protein structure is vital for maintaining cellular health^8^. The CCT5 structure harbors key functional domains, including a nucleotide-binding site, substrate-binding site, sensor loop, and apical loop, all of which are implicated in protein folding stability, a process linked to cancer pathogenesis^9^. Mutations in CCT5 have been associated with various adverse health outcomes, including distal and early onset motor neuropathy, chaperonopathy, hereditary sensory neuropathies, and various cancers, such as rectal cancer and non-small cell lung cancer (NSCLC)^10–14^.

Pan-cancer analysis is a powerful approach that integrates genomic, epigenomic, transcriptomic, and proteomic data from diverse cancer types and has immense potential for identifying commonalities and differences among various malignancies^15,16^. Information from pan-cancer analysis can be leveraged to develop targeted therapies and improve cancer diagnosis and treatment strategies^17^. Notably, previous pan-cancer analyses have been conducted for other CCT subunits, namely, CCT2, CCT3, and CCT8^18–20^. However, a comprehensive pan-cancer analysis specifically focusing on CCT5 is currently lacking, although recent studies have suggested a strong link between the entire CCT gene family and tumor proliferation^3,21^. Furthermore, evidence suggests a potential connection between CCT5 expression and various types of cancer, including lung, breast, gastric, esophageal, head and neck, and rectal cancers^8,13,22–27^. Therefore, a comprehensive pan-cancer analysis of CCT5 is warranted.

The Cancer Genome Atlas (TCGA) database provides an insightful resource for comprehensive pan-cancer analysis, making cancer bioinformatic analysis more efficient. TCGA offers a vast array of genomic data generated by high-throughput technologies such as next-generation sequencing, and TCGA empowers researchers to conduct in-depth analyses of cancer genome profiles^28^. Thus, this study aimed to investigate the potential role of CCT5 as a prognostic biomarker and therapeutic target across all 33 tumor types in TCGA database, thereby elucidating its oncogenic role in various cancers.

## Methods

### Sample information

**Fig. 1:**
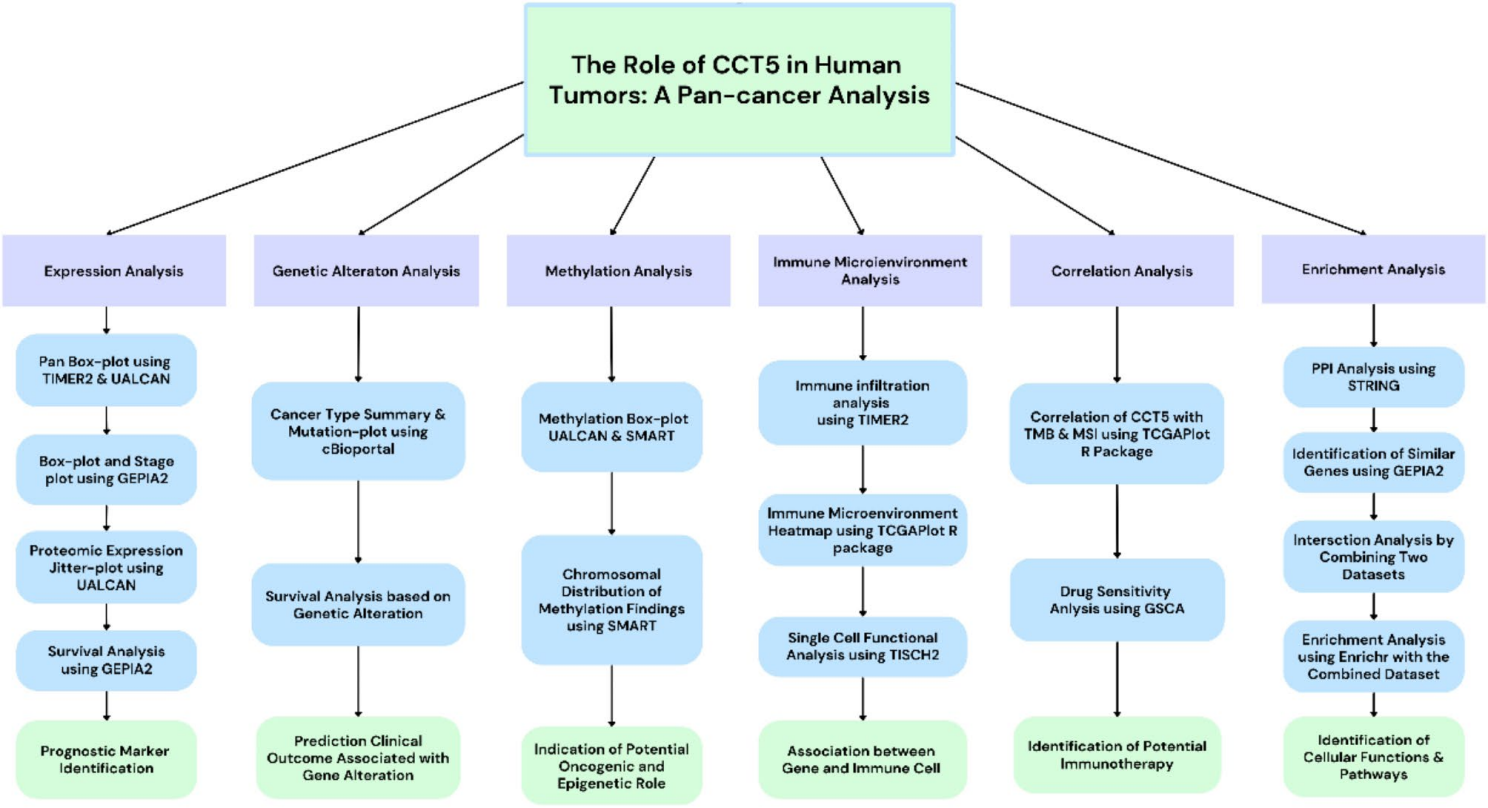
Schematic representation of the overall workflow of this study.

 The Cancer Genome Atlas (TCGA) (https://cancergenome.nih.gov/) provided the majority of the original data from public databases utilized for systematic pan-cancer analysis of CCT5. The full names and appropriate acronyms of the tumors are listed below: Adrenocortical carcinoma (ACC); Bladder Urothelial Carcinoma (BLCA); Breast invasive carcinoma (BRCA); Cervical squamous cell carcinoma and endocervical adenocarcinoma (CESC); Cholangiocarcinoma (CHOL); Colon adenocarcinoma (COAD); Lymphoid Neoplasm Diffuse Large B-cell Lymphoma (DLBC); Esophageal carcinoma (ESCA); Glioblastoma multiforme (GBM); Head and Neck squamous cell carcinoma (HNSC); Kidney Chromophobe (KICH); Kidney renal clear cell carcinoma (KIRC); Kidney renal papillary cell carcinoma (KIRP); Acute Myeloid Leukemia (LAML); Brain Lower Grade Glioma (LGG); Liver hepatocellular carcinoma (LIHC); Lung adenocarcinoma (LUAD); Lung squamous cell carcinoma (LUSC); Mesothelioma (MESO); Ovarian serous cystadenocarcinoma (OV); Pancreatic adenocarcinoma (PAAD); Pheochromocytoma and Paraganglioma (PCPG); Prostate adenocarcinoma (PRAD); Rectum adenocarcinoma (READ); Sarcoma (SARC); Skin Cutaneous Melanoma (SKCM); Stomach adenocarcinoma (STAD); Testicular Germ Cell Tumors (TGCT); Thyroid carcinoma (THCA); Thymoma (THYM); Uterine Corpus Endometrial Carcinoma (UCEC); Uterine Carcinosarcoma (UCS); Uveal Melanoma (UVM). The overall workflow for identifying and analyzing the role of CCT5 in human tumors is illustrated in Fig. 1.

### Gene expression analysis

The TIMER 2.0 (Tumor Immune Estimation Resource, version 2) database (http://timer.cistrome.org/) is an ideal tool for systematically examining the relationships between TCGA tumor characteristics and gene expression^29^. The “Gene DE” module of TIMER 2.0 was used to examine differences in CCT5 expression between tumor and normal tissues in multiple types of cancers. We again used the GEPIA2 ( Gene Expression Profiling Interactive Analysis, version 2) tool (http://gepia2.cancer-pku.cn) “Expression Analysis” module to validate the results of gene expression, whose data were derived from the Genotype-Tissue Expression (GTEx) and TCGA database^30^. The GEPIA2 tool provides box plots of gene expression between tumors and normal tissues. The expression of CCT5 in various clinical stages of tumors from TCGA database was examined using the GEPIA2 tool. Furthermore, using the UALCAN database (https://ualcan.path.uab.edu/), we examined differences in the expression levels of CCT5 in multiple types of cancer^31^.

### Proteomics analysis

The UALCAN (The University of Alabama at Birmingham Cancer data analysis Portal) database (https://ualcan.path.uab.edu/) was used to examine the differences in CCT5 protein expression between tumor and normal tissues. UALCAN provides a protein expression analysis option based on datasets from the International Cancer Proteogenome Consortium (ICPC) and the Clinical Proteomic Tumor Analysis Consortium (CPTAC). UALCAN provides a protein expression analysis of 14 tumor types, including colorectal cancer, breast cancer, ovarian cancer, clear cell renal cell carcinoma, uterine corpus endometrial carcinoma, gastric cancer, glioblastoma, pediatric brain tumors, head and neck squamous cell carcinoma, lung adenocarcinoma, lung squamous cell carcinoma, liver cancer, pancreatic cancer, and prostate cancer^32^.

### Survival analysis

The GEPIA2 (http://gepia2.cancer-pku.cn/) “Survival Analysis” module was used to generate overall survival (OS) and disease-free survival (DFS) plots and a survival significance map of CCT5 in all TCGA tumor types. The expression threshold was set at 50% for high and low CCT5 expression, and the median group cutoff. The prognostic value of CCT5 expression in 33 cancers was evaluated by disease-free survival (DFS) and overall survival (OS)^33^.

### DNA methylation analysis

We used the “TCGA analysis” module in the UALCAN database (https://ualcan.path.uab.edu/) to perform promoter methylation level analysis of CCT5 in normal tissues and cancers. The SMART website provides images of CpG-aggregated methylation and DNA methylation sites^34,35^.

### Gene alteration analysis

Genetic alteration data for CCT5, including alteration frequency, mutation type, and mutation site, were acquired from the cBioPortal database (v. 6.0.5) (https://www.cbioportal.org/), which is an open platform for analyzing cancer genomics data^33^. Based on datasets from TCGA Pan-Cancer Atlas Studies, we calculated the frequency of CCT5 mutation type of genes and alteration frequency in the “Cancer Types Summary” module. The “Mutations” module was used to generate a mutation site plot for CCT5.

### Immune infiltration analysis

“Immune” module of TIMER2 (http://timer.cistrome.org/) was employed to investigate the immune cell infiltration in which EPIC (Extended Polydimensional Immunome Characterization) algorithm is assessed. EPIC are correlated with CCT5 mRNA expression and cancer-associated fibroblast infiltration, CD8 + T cells, NK cell^29^.

### Correlation of CCT5 expression with TMB and MSI

Tumor mutation burden (TMB) is defined as the total number of mutations per million bases in the coding regions of gene exons. Additionally, it is an innovative biomarker for predicting the efficacy of immunotherapy in several tumor types, including bladder cancer, malignant melanoma, and lung cancer. Microsatellite instability (MSI) is a genetic alteration in the length of microsatellite sequences. During normal cell proliferation, a complete DNA mismatch repair system exists that detects replication errors in microsatellite sequences promptly and corrects them efficiently. This process enables high-fidelity replication of microsatellite sequences, thereby maintaining their stability. Due to deficiencies in DNA mismatch repair mechanisms during tumorigenesis, replication errors remain undetected, resulting in the insertion or deletion of repeated units or alterations in microsatellite sequence lengths, ultimately leading to microsatellite instability (MSI)^36^. The correlation between CCT5 expression and TMB or MSI was analyzed via Pearson correlation analysis, and a radar plot was generated by using the previously published TCGAplot (v8.0.0) R package (https://github.com/tjhwangxiong/TCGAplot)^37,38^.

### Pan-cancer correlation with CCT5 and immune microenvironment analysis

An analysis was conducted to investigate the correlation among CCT5 mRNA expression levels, immune cells, immune checkpoints, immune score, and immune regulatory genes (immunoinhibitory, immunostimulatory) utilizing the previously published TCGAplot (v8.0.0) R package (https://github.com/tjhwangxiong/TCGAplot).

### Single-cell functional analysis

Tumor Immune Single-cell Hub 2 (TISCH2) is a scRNA-seq database focusing on the tumor microenvironment (TME). TISCH2 gives a detailed description of different types of cells at the level of single cells, which helps us learn more about the tumor microenvironment (TME) in many types of cancer. We employed the “Gene Exploration” module in the TISCH2 database (http://tisch.comp-genomics.org/home/) to investigate the correlation between CCT5 and the TME at the single-cell level^39^. This module quantifies CCT5 expression in immune and stromal cells within the tumor microenvironment. The following principal parameters were defined: the gene named “CCT5”, the identification of cell type categorized as “Celltype (major-lineage)”, and the cancer types selected as BRCA, BLCA, CHOL, KIRC, LIHC, OV, STAD, SKCM, SARC, PAAD, UVM, and UCEC.

### Drug sensitivity analysis

To investigate the relationship between drug sensitivity and CCT5 mRNA expression, we analyzed drug screening data utilizing the GSCA (Gene Set Cancer Analysis) database (https://guolab.wchscu.cn/GSCA/#/) which integrates gene expression profiles for 33 cancer types from the TCGA, and drug sensitivity information from Genomics of Drug Sensitivity in Cancer (GDSC) and The Cancer Therapeutics Response Portal (CTRP) databases^40,41^.

### Gene enrichment analysis

STRING (*version*: *12.0*), a web-based tool (https://string-db.org/), was used to analyze the protein-protein interaction (PPI) network of the CCT5 gene. In this application, we configured parameters, such as meaning of network edges “Confidence”, active interaction sources “Textmining, Experiments, Databases, Co-expression, Gene Fusion, Neighborhood and Co-occurrence”, minimum required interaction score “low confidence 0.150” and maximum number of interactors to show “no more than 50 interactors” to retrieve the top 50 CCT5-related binding proteins. Following this analysis, the “Similar Gene Detection” module of GEPIA2 (http://gepia2.cancer-pku.cn) was employed to facilitate the identification of positively co-expressed genes. By employing the module of inquiry, the top 100 genes were selected from the TCGA normal and tumor datasets (Supplementary Data)^30,33^. Pearson correlation analysis was performed using the “Correlation Analysis” module of GEPIA2, in which the six significant genes were analyzed. To confirm the correlation analysis, we used the “Gene Corr” module of TIMER2.0 to generate a heatmap illustrating correlations and associated *p*-values and “Correlation Analysis” module of GEPIA2 to generate scatter plots. A Venn diagram was used to illustrate the intersected genes from CCT5 co-expressed and binding proteins using the InteractiVenn (Interactive Venn Diagrams) (https://www.interactivenn.net/) tool. Finally, the Enrichr database (https://maayanlab.cloud/Enrichr/) was used to identify the enriched functional categories and pathways of the binding protein and co-expressed genes^42^. KEGG, Reactome, and WikiPathway were the pathway enrichment analyses of CCT5, binding protein, and co-expressed genes, while the functional categories were Biological Process (BP), Cellular Component (CC), and Molecular Function (MF) of Gene Ontology^43–46^. We obtained permission to use KEGG pathway related figures in this study.

### Statistical analysis

The Wilcoxon test was used to evaluate the differential expression between tumors and adjacent normal tissues using TIMER 2.0. Analysis of Variance (ANOVA) and t-tests are primarily utilized in UALCAN as key statistical methods for assessing gene expression levels across different groups within cancer datasets. The correlation between gene expression was analyzed using Spearman’s correlation in GEPIA2. Statistical significance was set at *p* < 0.05. The alpha level for all the tests was set at 0.05.

### Ethics

All analyses were conducted using publicly available data to ensure compliance with the relevant guidelines and regulations.

## Result

### Expression level

**Fig. 2:**
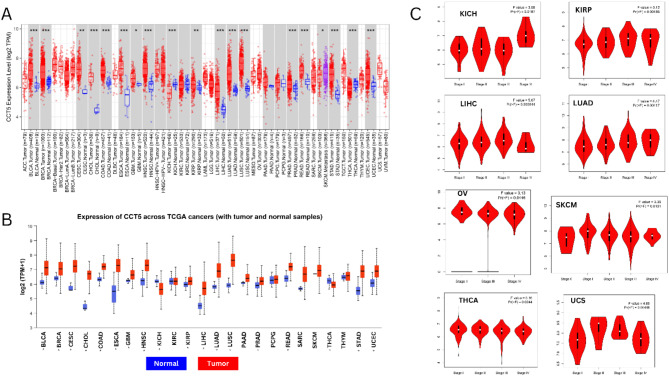
Expression analysis of CCT5 in different tumors and pathological stages. (**A**) Pan-cancer view of CCT5 between normal tissues and tumors via TIMER 2.0 (http://timer.cistrome.org/). (**B**) Pan cancer view of CCT5 across all TCGA tumor types analyzed using UALCAN (https://ualcan.path.uab.edu/). (**C**) CCT5 expression levels compared with different cancer stages including KICH, KIRP, LIHC, LUAD, OV, SKCM, THCA, and UCS using GEPIA2 (http://gepia2.cancer-pku.cn/).

We initially employed TIMER2.0, to analyze the expression levels of CCT5. As shown in Fig. 2A, the expression level of CCT5 was significantly higher in tumor cells than in the corresponding normal tissues, including BLCA, BRCA, CESC, CHOL, COAD, ESCA, GBM, HNSC, KIRP, LIHC, LUAD, LUSC, PRAD, READ, STAD, and UCEC, and was significantly downregulated in KICH, SKCM, and THCA. We further investigated the expression pattern of CCT5 in GEPIA2 in multiple cancer studies and compared it with that in normal tissues. This comparative analysis revealed upregulation of CCT5 in 19 different cancer types, namely BLCA, CESC, CHOL, COAD, DLBC, ESCA, GBM, LGG, LIHC, LUAD, LUSC, OV, PAAD, READ, SKCM, STAD, THYM, UCEC, and UCS, and its downregulation in LAML (Supplementary Fig. 1). Then, using the UALCAN database, we examined the variant expression levels of CCT5 in 24 cancer studies. As illustrated in Fig. 2B, CCT5 was significantly upregulated in 16 cancer types, including BLCA, BRCA, CESC, CHOL, COAD, ESCA, GBM, HNSC, KIRP, LIHC, LUAD, LUSC, PRAD, READ, STAD, and UCEC, and significantly downregulated in two distinct cancer types, KICH and THCA. Furthermore, it was noted that CCT5 expression in KICH, KIRP, LIHC, LUAD, OV, SKCM, THCA, and UCS (*p <* 0.05) was correlated with tumor stage using the GEPIA2 “stage plot” module Fig. 2C.

### Proteomic analysis

To enhance the consistency of CCT5 expression levels, the UALCAN database was used to analyze the proteomic expression of CCT5 in different tumors and normal tissues^47^. The expression of CCT5 in ovarian cancer, colon cancer, clear cell RCC, clear cell RCC (extended), UCEC, lung adenocarcinoma, lung squamous cell carcinoma, and head and neck squamous carcinoma was significantly higher than in normal samples. However, significantly lower proteomic expression has been observed in breast cancer, pancreatic adenocarcinoma, and glioblastoma multiforme. Statistical analysis revealed variations in CCT5 expression between the tumor and normal tissues, as shown in Supplementary Figure 2.

### Survival analysis

**Fig. 3:**
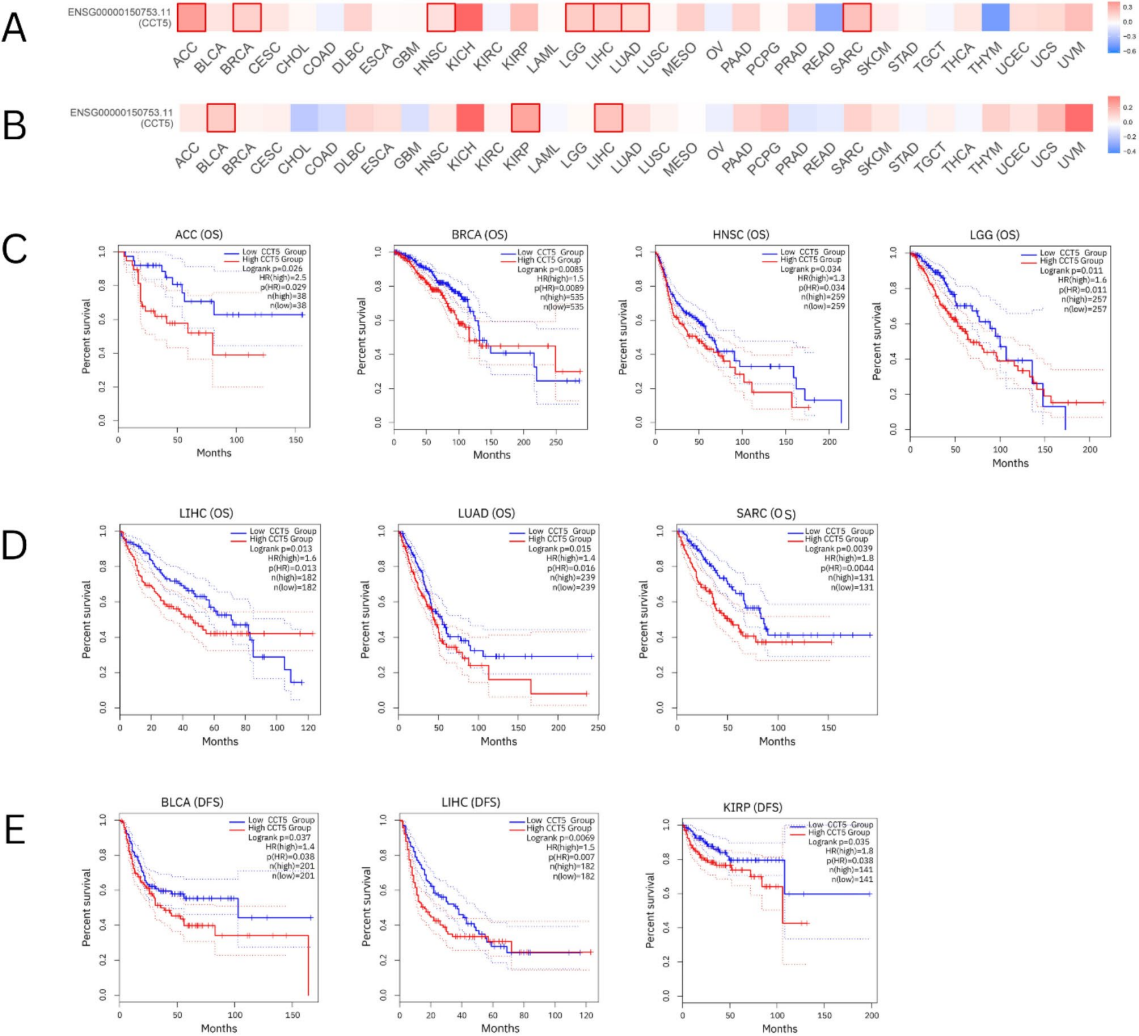
Survival analysis of CCT5 with high and low expression in different tumors was conducted using GEPIA2 (http://gepia2.cancer-pku.cn/). (**A**) The overall survival map showing CCT5 expression in different cancers. (**B**) Disease-free survival map showing CCT5 expression in different cancer types. (**C**) Overall survival curve of CCT5 expression in ACC, BRCA, HNSC, and LGG. (**D**) The overall survival curve for CCT5 expression in LIHC, LUAD, and SARC. (**E**) Disease free survival curve of CCT5 expression in BLCA, LIHC and, KIRP (*p* < 0.05).

To explore the potential prognostic value of CCT5 based on TCGA database, we investigated the correlation between CCT5 expression and prognosis of patients with different tumors using GEPIA2. Higher CCT5 was associated with shorter overall survival (OS) in patients with ACC (log-rank *p* = 0.026), BRCA (log-rank *p* = 0.0085), HNSC (log-rank *p* = 0.034), LGG (log-rank *p* = 0.011), LIHC (log-rank *p* = 0.013), LUAD (log-rank *p* = 0.015), and SARC (log-rank *p* = 0.0039). Furthermore, disease-free survival (DFS) analysis showed that high CCT5 expression was a marker for poor outcomes in patients with BLCA (log-rank *p* = 0.037), KIRP (log-rank *p* = 0.035), and LIHC (log-rank *p* = 0.0069) (Fig. 3).

### Methylation analysis

To examine the potential association between DNA methylation of CCT5 and the formation of diverse tumor types, methylation analysis was performed. We utilized the UALCAN online resources to investigate the promoter methylation level of CCT5 in various types of cancer. The promoter methylation levels of CCT5 were downregulated in BLCA, BRCA, CESC, CHOL, COAD, ESCA, HNSC, KIRP, KIRC, LIHC, LUAD, LUSC, PAAD, PRAD, READ, SARC, TGCT, THCA, and UCEC and were comparable between tumor and normal tissues (*p* < 0.05). (Supplementary Fig. 3)

**Fig. 4:**
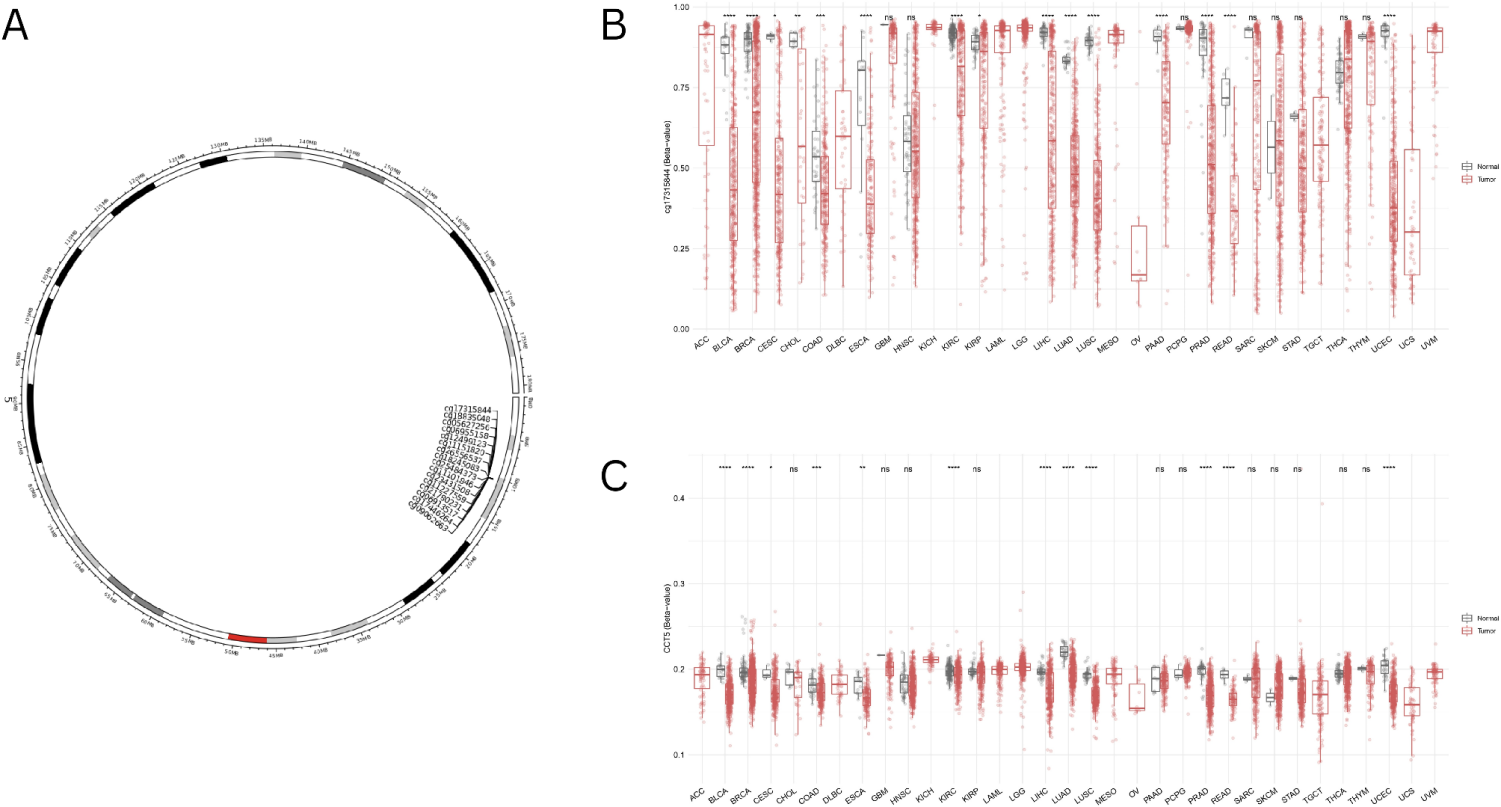
Methylation analysis of CCT5 in different tumors. (**A**) Images of chromosomal distribution of methylation probes associated with CCT5. (**B**) The differences in the methylation levels of CCT5 between the tumor and healthy groups are graphically illustrated using boxplots that demonstrate the upregulation or downregulation in tumors (red) as opposed to healthy tissues (gray) across a variety of cancer types. This pattern was observed using the N-shore probe cg17315844. Beta values vary between 0 and 1, where 0 indicates the absence of methylation and 1 signifies full methylation. Statistical significance determined by the Wilcoxon test is denoted by the number of stars (*p < 0.05, **p < 0.01, ***p < 0. 001, ****p < 0.0001). (**C**) Analysis of CpG-aggregated methylation of CCT5 in human cancers with SMART.

Furthermore, we conducted extensive research on the methylation of CCT5 across numerous cancer studies, employing the SMART database (an interactive web application for comprehensive DNA methylation analysis and visualization) to compare methylation levels in cancerous tissues to those in normal tissues. The plot from the SMART database shows the chromosomal distribution of the methylation probes associated with CCT5 (Fig. 4A). Analysis of 16 probes from the SMART database was conducted across pan-cancers to investigate the methylation status of CCT5. The N-shore probe cg17315844 revealed a pattern of reduced methylation in nearly all malignancies examined compared to healthy tissues (Fig. 4B). The CpG-aggregated methylation analysis of CCT5 in human cancers demonstrated that the methylation level of CCT5 was lower in BLCA, BRCA, CESC, COAD, ESCA, KIRC, LIHC, LUAD, LUSC, PRAD, READ, and UCEC (Fig. 4C).

### Gene alteration analysis

**Fig. 5:**
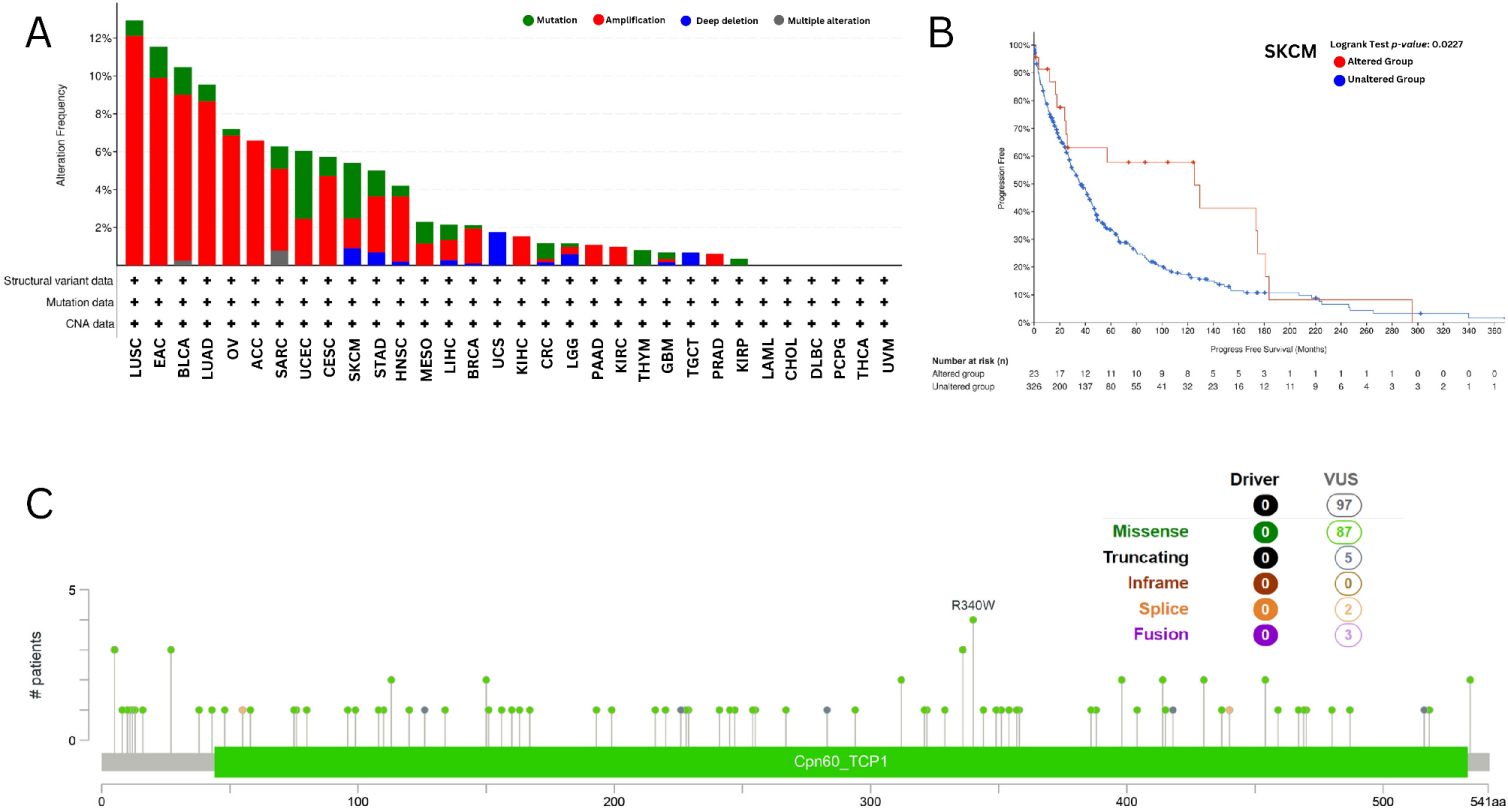
Genetic alteration analysis of CCT5 using cBioportal (https://www.cbioportal.org/). (**A**) Cancer type summary for CCT5 gene. (**B**) Progression-free survival curve for SKCM (*p* = 0.0227). (**C**) Mutation plot for CCT5.

Using cBioportal, as illustrated in Fig. 5A, we examined CCT5 genetic alteration status in human malignancies in TCGA cohorts. We discovered that the highest frequency of gene alterations (> 10%) was observed in the amplification alterations present in the LUSC tumor samples. Furthermore, CCT5 genetic alterations were greater than 8% in the EAC (Esophageal Adenocarcinoma), BLCA, and LUAD samples. In addition, the highest mutation alteration was found in UCEC and a deep deletion alteration was observed in UCS. Figure 5C illustrates the total number of CCT5 mutations found in TCGA samples, which included 87 missense mutations, five truncating mutations, two splice mutations, and three fusion mutations. In this analysis, we observed that the main genetic alteration type was amplification, and the amplification alteration of R340W was found in the Cpn60_TCP1 domain in one case of UCEC and STAD and two cases of CESC. Furthermore, we investigated the association between CCT5 genetic alterations and clinical outcomes in cancer patients. The CCT5 mutation was associated with better prognosis in SKCM patients in terms of progression-free survival (PFS) (*p* = 0.0227) (Fig. 5B).

### Immune infiltration analysis

**Fig. 6:**
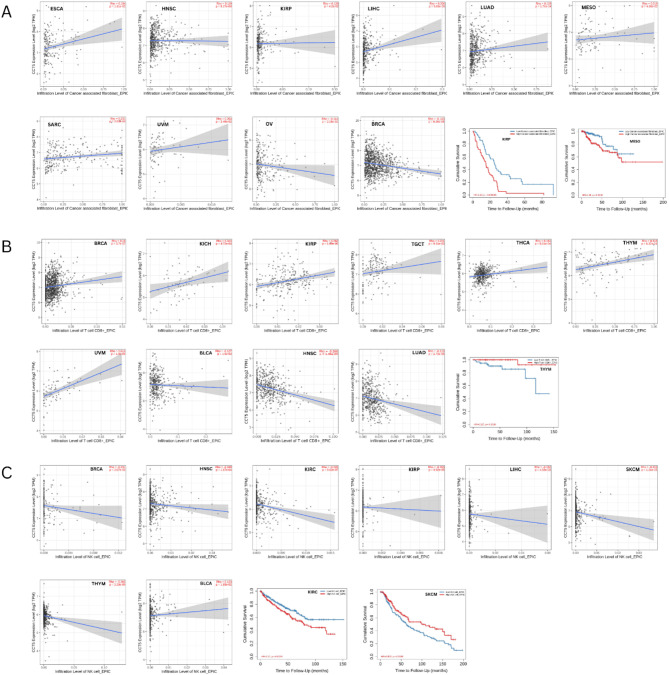
Correlations between CCT5 expression with (**A**) cancer associated fibroblast, (**B**) CD8 + Tcell, and (**C**) NK cell immune infiltration level and survival curves, respectively.

Prior research has revealed that stroma-associated cancer-related fibroblasts play a role in the control of various immune cells that invade tumors^48^. NK and CD8 + T cells exhibit antitumor activities in innate and adaptive immunity, respectively^49,50^. Therefore, we used EPIC algorithms to examine the relationship between CCT5 expression in various cancers and cancer-associated fibroblasts, CD8 + T cells, and NK cell infiltration. Cancer-associated fibroblast infiltration in ESCA, HNSC, KIRP, LIHC, LUAD, MESO, SARC, and UVM positively correlated with CCT5 expression. However, BRCA and OV were negatively correlated. Significant survival curves were associated with MESO (*p* = 0.000695) and KIRP (*p* = 0.0149) for CAFs infiltration (Fig. 6A).

In addition, CCT5 and CD8 + T-cell infiltration in BRCA, KICH, KIRP, TGCT, THCA, THYM, and UVM showed a positive correlation, and negative correlations were found in BLCA, HNSC, and LUAD, whereas significant survival analysis was associated with THYM (*p* = 0.0308) (Fig. 6B).

For NK cell infiltration, a significant positive correlation was observed with BLCA and negatively correlated with BRCA, HNSC, KIRC, KIRP, LIHC, SKCM, and THYM. Survival prognosis was significantly associated with SKCM (*p* = 0.0138) and KIRC (*p* = 0.0129) (Fig. 6C).

### Correlation of CCT5 expression with TMB and MSI

**Fig. 7:**
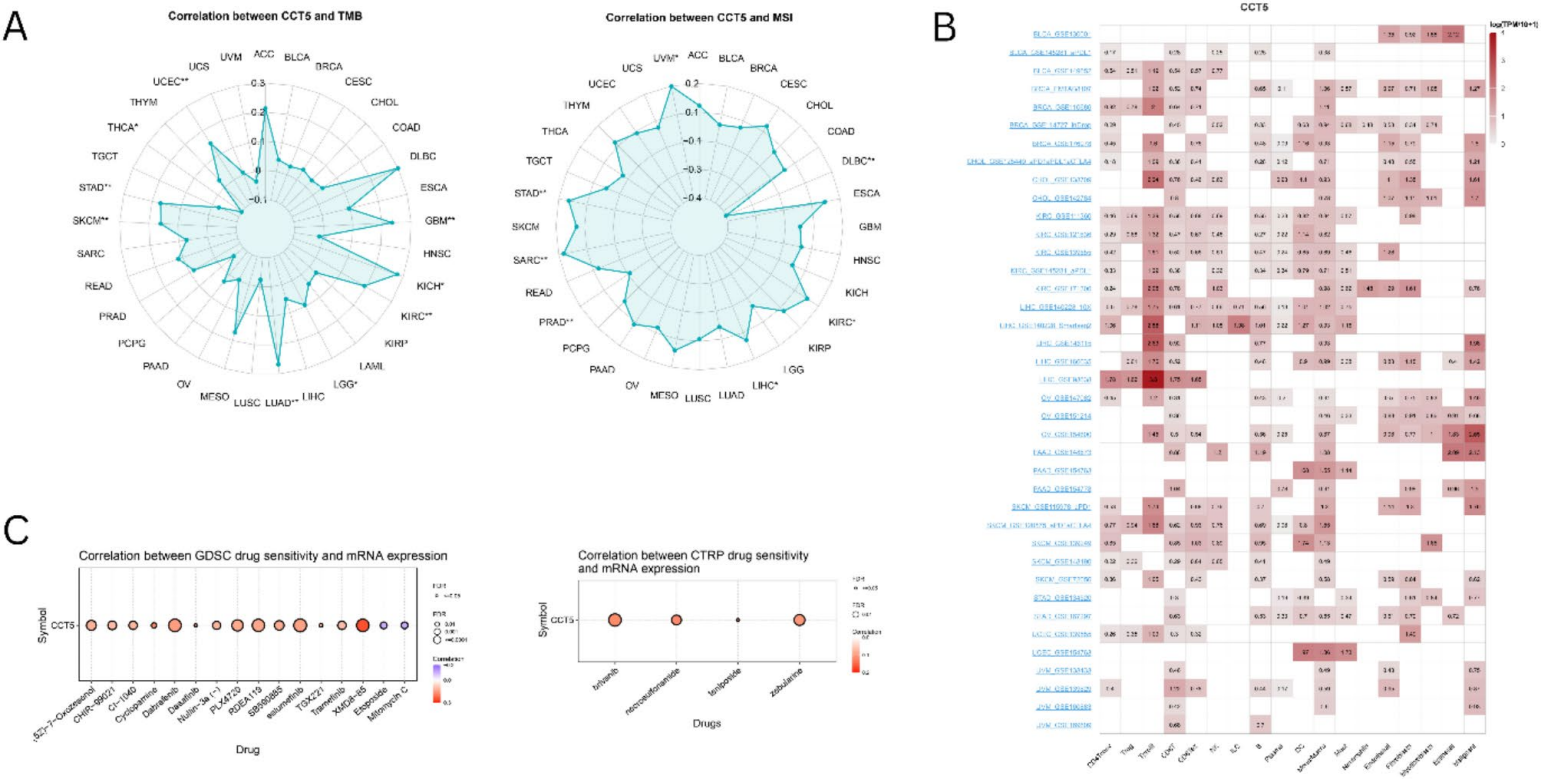
Pan-cancer correlation analysis of CCT5 expression with TMB and MSI, and drug sensitivity. Pan-cancer expression analysis of CCT5 from single cell RNA sequencing data. (**A**) Pan-cancer correlation analysis of CCT5 with TMB and MSI, (**B**) Pan-cancer expression analysis of CCT5 in immune cell subgroups from scRNA sequencing data, (**C**) The correlation between CCT5 expression and the sensitivity to top GDSC and CTRP drugs in pan-cancer was examined.

Correlation analysis revealed that CCT5 expression was significantly associated with TMB and MSI in various cancers. CCT5 expression exhibited a significant positive correlation with TMB in patients with GBM, KICH, KIRC, LGG, LUAD, SKCM, STAD, and UCEC, and a significant negative correlation with THCA (*p < 0.05, **p < 0.01). Additionally, CCT5 expression showed a significant positive correlation with MSI in KIRC, LIHC, SARC, STAD, and UVM, and a significant negative correlation with MSI in DLBC and PRAD (*p < 0.05, **p < 0.01) (Fig. 7A). These data demonstrated that CCT5 exhibited a significant correlation with TMB and MSI in KIRC and STAD. Consequently, CCT5 may influence antitumor immunity through its association with TMB and MSI in KIRC and STAD.

### Pan-cancer correlation with CCT5 and immune microenvironment

Immune and stromal cells play crucial roles in the regulation of the development and progression of cancers, constituting the major components of the tumor microenvironment (TME), and their infiltration levels influence the efficacy of immunotherapy^37^. CCT5 was strongly negatively associated with immune scores in STAD, LUAD, SKCM, LUSC, UCEC, GBM, TGCT, BRCA, COAD, and PRAD (Supplementary Fig. 4C). In most tumor samples across the 33 types of tumors, CCT5 was positively correlated with Macrophages M0, Macrophages M1, Macrophages M2, resting memory CD4 T cells in most tumors, while was negatively associated with T cell regulatory Tregs, activated NK cells, Plasma cells, memory B cells, and CD8 T cells (Supplementary Fig. 4E). CCT5 exhibited positive associations with immune checkpoint-associated genes in BRCA, BLCA, KIRP, KICH, KIRC, LIHC, PCPG, PRAD, STAD, OV, UVM, and UCEC (Supplementary Fig. 4A). CCT5 demonstrated positive correlations with almost all immune inhibitory or stimulating genes in BLCA, LIHC, LGG, KICH, KIRC, KIRP, PCPG, PRAD, PAAD, and UVM, while displaying negative correlations in LUSC, ESCA, and LUAD (Supplementary Fig. 4B,D). These collective data suggest that CCT5 expression exhibited widespread correlation with immunity in cancers and may influence survival through interactions with immune infiltration. This correlation could be either negative or positive, contingent upon the specific tumor type.

### Single-cell functional analysis

The tumor microenvironment comprises a heterogeneous collection of cells, including immune, stromal, and cancer cells. We used the TISCH2 database to investigate CCT5 expression in different cell types within the tumor microenvironment. CCT5 demonstrates higher expression levels in immune cells, particularly Tprolif (Proliferating T cell), CD4Tconv (Conventional CD4 T cells), Treg (Regulatory T cells), CD8T (CD8 T cells), CD8Tex (Exhausted CD8 T cells), NK cells, B cells, Plasma cells, DC (Dendritic cells), and Mono/Macro (Monocytes or macrophages), compared with stromal cells like fibro blasts, endothelial cells, and myofibroblasts, in most cancers (Fig. 7B).

### Drug sensitivity analysis

The correlation between CCT5 and drug sensitivity in the CTRP and GDSC databases was analyzed using the Gene Set Cancer Analysis database. Our analysis revealed a positive correlation between CCT5 expression and drug sensitivity in the GDSC and CTRP databases for several compounds, including XMD8 − 85 (ERK5 inhibitor), Dabrafenib (kinase inhibitor), RDEA119 (MEK1/2 inhibitor), Selumetinib (MEK 1/2 inhibitor), PLX4720 (B-RafV600E inhibitor), (5Z) − 7−Oxozeaenol (TAK1 inhibitor), SB590885 (B-Raf kinase inhibitor), Trametinib (MEK inhibitor), CHIR − 99,021 (GSK-3β Inhibitor), CI − 1040 (MEK1/MEK2 inhibitor), Nutlin − 3a (p53-MDM2 inhibitor), Cyclopamine, Dasatinib, TGX221(PI3K inhibitor), Brivanib (VEGFR2 inhibitor), Zebularine (DNA methyltransferase inhibitor), and Necrosulfonamide (necroptosis inhibitor). Conversely, a negative correlation was observed between CCT5 expression and sensitivity to two drugs, namely Etoposide (topoisomerase II inhibitor) and Mitomycin C (Fig. 7C). These findings suggest that CCT5 may be a useful target for cancer treatment.

### Gene enrichment analysis

**Fig. 8:**
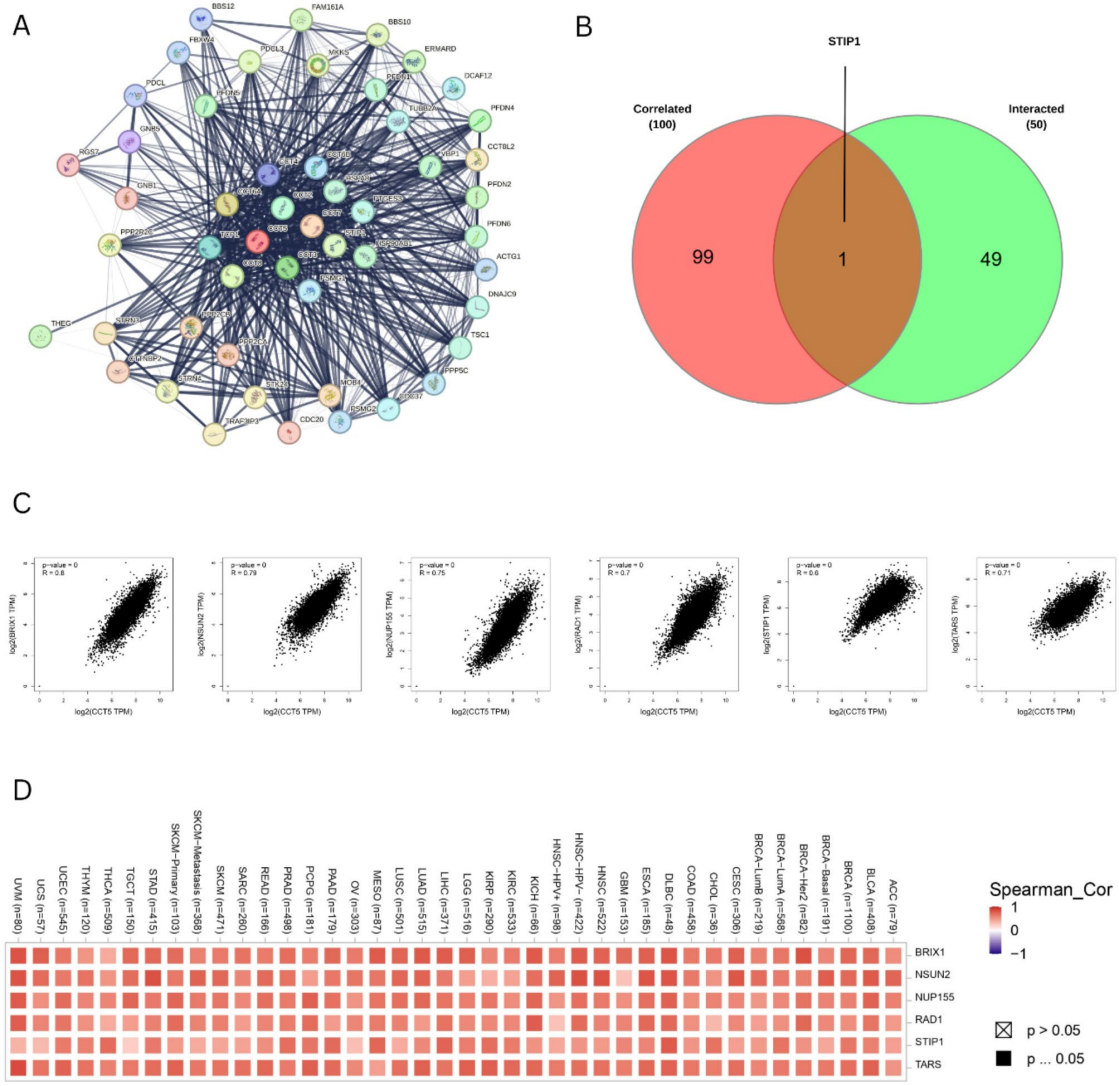
CCT5-related co-expressed genes analysis. (**A**) The CCT5-binding proteins (PPI) identified utilizing the STRING tool, (**B**) Venn diagram illustrating the intersection analysis of the CCT5-binding and correlated genes, (**C**) The correlation between the expression of CCT5 and the six co-expressed genes with CCT5 (BRIX1, NUN2, NUP155, RAD1, TARS, and STIP1), (**D**) Heatmap depicting the correlation between the expression of CCT5 and the six co-expressed genes with CCT5 (BRIX1, NUN2, NUP155, RAD1, TARS, and STIP1) in the multiple cancer types.

**Fig. 9:**
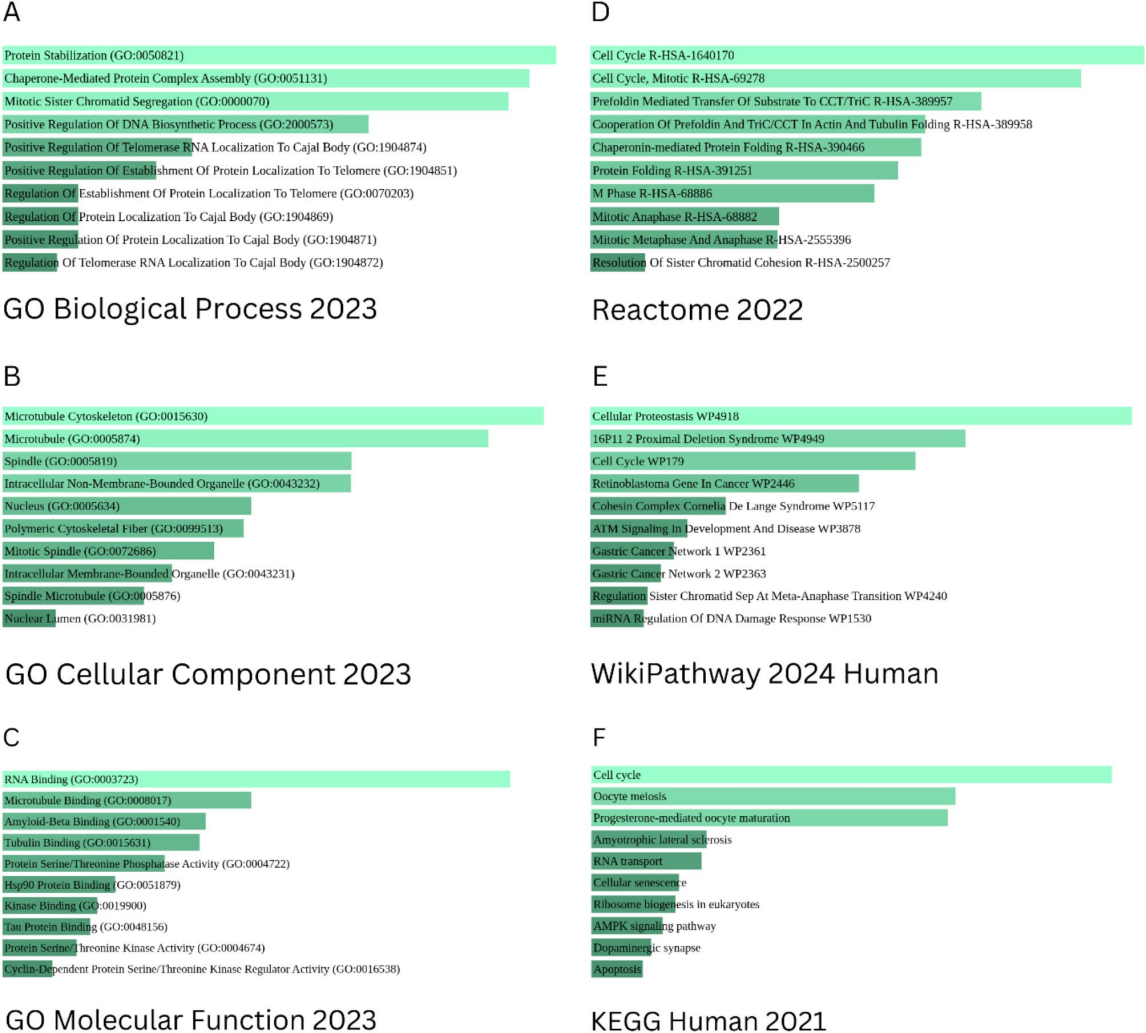
CCT5-related gene enrichment analysis using Enrichr (https://maayanlab.cloud/Enrichr/). The bar diagrams indicate a significant association between CCT5 and different metabolic pathways, and a lighter color indicates a more significant result. (**A**) GO Biological Process 2023, (**B**) GO Cellular Component 2023, (**C**) GO Molecular Function 2023, (**D**) Reactome Pathway 2022, (**E**) Wiki Pathway 2024 Human, and (**F**) KEGG Human 2021.

To assess tumorigenesis of CCT5, 50 binding proteins of CCT5 and 100 co-expressed genes were obtained using STRING and GEPIA2, respectively. The combined results were used for carry out a gene enrichment analysis of CCT5. Figure 8A presents the results of the protein-protein interaction network of CCT5. In addition, 100 top most positively co-expressed genes were obtained, and six genes were selected for further validation, including BRIX1, NSUN2, NUP155, RAD1, STIP1, and TARS. The results, as shown in Fig. 8C, indicate the strong positive correlation between BRIX1 (*R* = 0.8), NSUN2 (*R* = 0.79), NUP155 (*R* = 0.75), RAD1 (*R* = 0.7), STIP1 (*R* = 0.6), and TARS (*R* = 0.71) along with CCT5 expression. The results obtained from the heatmap analysis also revealed positive correlations between those six genes and CCT5 expression across all cancers (Fig. 8D). The Venn diagram provides the intersection analysis, illustrating a common member STIP1 among the binding proteins and co-expressed genes (Fig. 8B). Finally, GO Biological Process suggested the involvement of CCT5 with “Protein Stabilization”, “Chaperone-mediated protein complex assembly”, and “Mitotic sister chromatid segregation”. GO analysis of the Molecular Function revealed a connection between CCT5 and “RNA binding,” “Microtubule binding”, “Amyloid-beta binding” “Tubulin binding” and, “Protein serine/threonine phosphatase activity”. In addition, GO Cellular Component 2023 demonstrated the interaction of CCT5 with “Microtubule cytoskeleton”, “Microtubule”, “Spindle”, “Intracellular Non-membrane-bound organelles”, and “Polymeric cytoskeletal fiber”. The Reactome 2022 pathway revealed that the CCT5 plays a role in the cell cycle (*p-value = 2.982e-32*, odds ratio = 14.13). WikiPathway 2024 Human showed the connection of CCT5 with “Cellular proteostasis WP4918” (*p-value = 1.545e-13*, odds ratio = 119106.00), “16P11 2 proximal deletion syndrome WP4949” (*p-value = 1.856e-10*, odds ratio = 22.24), “Cell cycle WP179” (*p-value = 1.566e-9*, odds ratio = 14.44), and “Retinoblastoma Gene in Cancer WP2446” (*p-value = 1.734e-8*, odds ratio = 16.30). KEGG 2021 human enrichment analysis revealed that CCT5 was interconnected with the cell cycle (*p-value = 1.455e-10*, odds ratio: 15.44) (Fig. 9A,F) (Supplementary Tables 1–6).

## Discussion

The Cancer Genome Atlas (TCGA) study identified 33 prevalent tumor types using multi-omics data and provided a unique opportunity to identify molecular abnormalities across all types of cancer^33^. Chaperone proteins are a family of proteins that are classified into heat shock protein 60 and TCP-1 chaperone protein complex. TCP-1 ring-containing chaperone protein complex (TRiC) production requires the presence of a CCT molecule. This complex consisted of two identical stacked rings, each containing eight different proteins^18^. CCT5 is a subunit of chaperonin that contains the TCP1 complex, also known as the TCP1 ring complex (TRiC)^23^.

We analyzed the prognostic value and oncogenic role of CCT5 in multiple types of cancers. According to the Ensembl database, CCT5 is located on chromosome 5p15.2 and its gene synonym is KIAA0098. This gene has 15 transcripts, 219 orthologs, and 13 paralogs and is associated with two phenotypes. A recent study showed that tumor proliferation is closely related to the CCT family of genes^18^. However, the significance of CCT5 in various tumor types has not been extensively explored. Consequently, we comprehensively examined characteristics such as gene expression, proteomic expression, DNA methylation, survival analysis, genetic alteration, immune infiltration, and pathway analysis to characterize CCT5 in 33 TCGA tumor types.

CCT5 is highly expressed and upregulated in most tumors, indicating its tumor driver potency. We further investigated the relationship among CCT5 overexpression, clinical parameters, and prognosis. Survival analysis revealed that CCT5 overexpression was associated with poor OS and DFS. High CCT5 expression is associated with a poor prognosis in different tumor types, including HNSC, LUAD, BLCA, and KIRP. As increased expression of CCT5 indicates poor prognosis, it could be possible to increase the survival rate by reducing its expression^51^. In addition, there was a significant relationship between increased CCT5 expression and advanced cancer stage, indicating invasive development.

DNA methylation plays an important role in epigenetics. Moreover, epigenetic alterations are vital for carcinogenesis progression. DNA methylation is commonly described as a ‘silencing’ epigenetic modification that plays a crucial role in enhancing the stability of transcriptional repression^52^. Most DNA methylation is essential for normal development; however, it is dysregulated and contributes to disease like cancer^53^. DNA methylation is a potential biomarker for the evaluation and prediction of cancer outcomes. The regulation of gene expression related to tumors in individuals with the disease is significantly influenced by DNA methylation^54^. According to the results of this study, CCT5 was highly expressed in tumor tissues and was associated with a poor prognosis; hence, it was postulated that CCT5 could potentially function as an oncogene. Compared to the corresponding normal tissues, the promoter methylation level of CCT5 in tumor tissues was significantly reduced, and the outcome was a reduction in the stability of transcriptional repression, consequently resulting in an elevation of CCT5 expression levels and promotion of tumor progression. Furthermore, the N-shore probe cg17315844 revealed a pattern of reduced methylation in nearly all malignancies examined in comparison to healthy tissues. This suggested that differential methylation of the CCT5 N-shore site may play a role in pan-cancer carcinogenesis^55^. These findings suggest that CCT5 may possess the ability to regulate specific tumors at the epigenetic level.

Some studies have suggested that genetic alterations affect tumor development and treatment effectiveness. For example, genetic alterations in BRCA1 and BRCA2 significantly correlate with patient survival^56,57^. The oncogenic role of a gene in a particular cancer type can be assessed by genetic alteration analysis. In this study, we found that mutations in CCT5 are most common in LUSC (> 10%), followed by Esophageal Adenocarcinoma (EAC), Bladder Urothelial Carcinoma (BLCA), and Lung Adenocarcinoma (LUAD), which cumulatively indicates that CCT5 mutations influence cancer progression in different tissues. To determine whether genetic changes in CCT5 affect clinical outcomes in patients with cancer, we determined that the CCT5 mutation may be a protective factor for patients with skin cutaneous melanoma (SKCM). According to a recent study, approximately 75-80% of skin cancer-related deaths each year are caused by SKCM, which suggests that SKCM is one of the most aggressive cancers^58,59^. In addition, these findings indicate that CCT5 acts as an oncogene in cancer development and may be an indicator for use in clinical applications in cancer prognosis.

Immune cells intricately engage with cancer cells and exert a significant influence on the migration and propagation of cancer within different tumor categories^60^. Recent studies have also shown a correlation between the tumor immune microenvironment and expression levels of various genes^61,62^. In this study, the expression of CCT5 was positively correlated with the infiltration of cancer-associated fibroblasts (CAFs) in numerous tumor types. In the context of various types of malignancies, cancer-associated fibroblasts (CAFs) are key stromal cells that have been shown to correlate with unfavorable prognosis, resistance to chemotherapy, and disease revival^63–66^. The significant survival curves for MESO and KIRP revealed that high-level infiltration of CAFs was associated with poor prognosis. CD8 + T cells play an important role in cancer immunity^50^. A significant positive correlation between CCT5 and CD8 + T cells has been observed in most cancer types; however, several studies have shown a negative correlation. Furthermore, low CD8 + T-cell infiltration may decrease the survival rate of THYM patients. We also evaluated the correlation between CCT5 and NK cells and found that most cancer types demonstrated a negative correlation. A negative correlation indicates that when CCT5 expression is higher in the tumor microenvironment, the NK cell infiltration level remains low. Survival prognosis also provides meaningful insight that is statistically significant for SKCM and KIRC, describing the poor prognosis of low-level NK cell infiltration and high-level NK cell infiltration, respectively. Our study emphasizes the role of CCT5 in providing prognostic information for various cancer types. In this study, the expression of these three tumor-infiltrating immune cells was significantly correlated with the prognosis of patients with MESO, KIRP, THYM, KIRC, and SKCM. Our observations compel us to propose that CCT5 may play a role in regulating the level of tumor-infiltrating immune cells, consequently affecting the prognosis of these tumors. Therefore, CCT5 could be a potential target for immunotherapy.

Immunotherapy has emerged as a potentially effective treatment approach for various cancers, with both MSI and TMB serving as crucial biomarkers of immunotherapeutic response^37,67^. Previous studies have demonstrated that these two indicators can predict patient responses to multiple therapeutic agents or drugs, particularly immune checkpoint inhibitors^36^. In this investigation, we observed that CCT5 expression was associated with TMB in 9 cancer types and with MSI in 7 cancer types. CCT5 exhibited a positive correlation with both TMB and MSI in KIRC and STAD. Consequently, we hypothesized that these tumor types may benefit from immunotherapy.

The tumor microenvironment (TME) plays a critical role in tumor proliferation, invasion, and drug resistance and comprises both stromal and immune cells^37^. In this study, CCT5 was strongly negatively associated with immune score in 10 types of cancer. Furthermore, CCT5 was positively correlated with invasive immune cells in most tumors, including Macrophages and CD4 T cells, while negatively correlated with regulatory T (TReg) cells, NK cells, B cells and CD8 T cells. CD4 T cells enhance the antitumor activity of CD8 T cells and macrophages, consequently inhibiting tumor growth. Regulatory T cells (TReg cells) play a crucial role in preventing autoimmunity, maintaining peripheral tolerance, and attenuating chronic inflammatory diseases under normal conditions. However, regulatory T cells (TReg cells) inhibit sterilizing immunity, anti-tumor immunity, promote tumor growth, facilitate immune escape, and suppress beneficial responses to immunotherapy during tumor development and progression^37^. These findings suggest that the expression of CCT5 was associated with immune cell infiltration and their immunological functions in the tumor microenvironment (TME). The use of immune checkpoint inhibitors is one of the key approaches in immune-therapy. Immune checkpoints typically suppress the body’s immune response against healthy cells. Certain malignancies can evade these regulatory checkpoints, thereby preventing the immune system from identifying tumor cells. Therefore, blocking these checkpoints makes it possible to identify cancer cells and trigger an immune response against them. Patients with advanced cancer may have a higher chance of survival if they take immune checkpoint inhibitors. These include lung cancer, urothelial bladder cancer, renal cell carcinoma, and melanoma^67^. In this study, we found that CCT5 was positively associated with immune checkpoint genes in 12 types of cancers, and a similar correlation was found in immune stimulating and inhibitory genes.

We also explored the expression of CCT5 at single cell level in TISCH2 database. CCT5 demonstrates higher expression levels in immune cells, compared with malignant and stromal cells across several types of tumors. These data suggest that CCT5 may regulate the immune response by influencing the expression of immune-related genes.

The results of the drug sensitivity test from the GSCA tool showed that XMD8-85, Selumetinib, RDEA119, Dabrafenib, and Brivanib are the most sensitive drugs associated with CCT5 expression. Selumetinib is a drug that has effects on the inhibition of various protein kinase pathways, which leads them to be MEK1/2 (mitogen-activated kinase kinase) inhibitor^68^. For treating thyroid cancer, selumetinib can reverse radioiodine resistance, making it a potential therapeutic option^68^. Studies have suggested that dabrafenib and RDEA119 can act as targeted therapies for pancreatic cancer^69,70^. Brivanib is a novel VEGF2 inhibitor that is a major family of angiogenic growth factors that facilitate tumor growth during early angiogenesis^71^. As CCT5 expression is associated with the sensitivity of these drugs, this investigation suggests that it may help to develop targeted immunotherapy in the future for any cancer associated with the expression of CCT5.

Furthermore, we identified genes that were positively co-expressed with CCT5 and performed a validation analysis that revealed positive results. The venn diagram shows the intersected gene of CCT5, which is positively co-expressed and acts as a binding protein. Enrichment analysis using Enrichr, a web-based tool, was performed using the combined genes. The enrichment analysis indicated that CCT5 was significantly associated with various molecular functions and signaling pathways. Based on Gene Ontology, CCT5 and its co-expressed genes exhibit associated functions in protein stabilization, chaperone-mediated protein complex assembly, mitotic sister chromatid segregation, cytoskeleton organization, RNA binding, microtubule binding, amyloid-beta binding, and protein serine/threonine phosphatase activity, and any dysfunction in these functions may influence tumorigenesis. Previous studies have indicated that RNA-binding proteins exhibit diverse mechanisms, including polyadenylation, subcellular localization, and translation, and that malfunctioning of these mechanisms may influence cancer metastasis^72,73^. Amyloid-beta precursor protein is involved in induction of p27^kip1^ and caspase-3 mediated apoptosis that results the blocked of cell division^74^. Knockdown of this protein may delay cells in G1 phase that eventually prevents cells from moving to the S phase of the cell cycle, resulting in a reduced S phase population of cell^74^. A previous study suggested that amyloid-beta immunoreactivity is concentrated in blood vessels, and their systemic production induces tumor accumulation^75^. Thus, the molecular function of amyloid beta binding activity of CCT5 and the co-expressed genes may influence tumorigenesis. Cytoskeletal molecules, including microtubules, play roles in both normal and cancer cells. Microtubules and tubulins regulate cellular homeostasis^76^. Despite differences in the expression of microtubule-associated proteins (MAP) and tubulins involved in chemoresistance, microtubule-associated chemotherapeutic agents have been developed to address drug resistance^76^. In chemotherapeutics, microtubules and tubulins are highly recognized therapeutic targets because of their homeostatic functions in cellular processes, including stress response, mitotic spindle dynamics suppression, and p53 activity regulation^77^. The contribution of CCT5 to cytoskeletal microtubule function requires further exploration for the potential development of chemotherapeutic interventions. Protein serine/threonine phosphatase activity regulates numerous cellular processes, including embryonic development, cell proliferation, cell death, and cancer^78^. These findings suggest that of CCT5 contributes to cellular processes, and aberrant expression can lead to the impairment of cellular processes, which eventually causes cancer. KEGG, WikiPathway, and Reactome also provided meaningful insights into CCT5 and its co-expressed genes, and these three databases exhibited the association of CCT5 with the cell cycle pathway. Cell cycle dysfunction is associated with malignant growth, leading to cancer development^79,80^. It is a fundamental biological pathway, and the entire cellular process relies on this pathway. Cancer proliferation primarily relies on the failure of the exit of the cell cycle, causing continuous division. Studies suggest that cell cycle checkpoints prevent the genetic malfunction of cell growth, and persistent cell growth, which is regulated by mutations that promote the prevention of apoptosis, is considered a hallmark of cancer^81,82^.

This study has numerous limitations. To analyze the potential role of CCT5 in pan-cancer, we used different publicly available databases that derived original data from TCGA databases. However, the data collection and processing methods may not be consistent across databases, which may introduce systematic biases. Limited numbers of samples for certain uncommon tumor types may have led to inaccurate results, such as UALCAN containing insufficient amounts of normal sample for multiple types of tumor in expression, proteomic, and methylation analyses. Additionally, this study used only a bioinformatics approach that provided preliminary evidence linking CCT5 with cancer progression in various tumors. Further in vivo or in vitro experimental research, such as RT-qPCR (real-time quantitative polymerase chain reaction) and WB (western blotting) is necessary to validate the results and clarify the specific molecular role of CCT5 at the cellular and molecular levels in carcinogenesis.

## Conclusion

Our comprehensive pan-cancer analysis of CCT5 revealed that it is widely overexpressed in various cancer types, and that its expression and genetic alterations are significantly associated with clinical outcomes in patients with certain tumors. Survival prognosis indicated that CCT5 may act as a potential biomarker for several cancer types. According to the methylation analysis of this study, it was postulated that CCT5 could potentially function as an oncogene and may possess the ability to regulate specific tumors at the epigenetic level. Furthermore, our findings suggest that CCT5 expression correlates with immune cell infiltration and may have a potential role in regulating the number of tumor-infiltrating immune cells. CCT5 exhibited a significant correlation with TMB and MSI in KIRC and STAD. Single-cell analysis revealed higher CCT5 expression in immune cells than in malignant and stromal cells across several types of tumors. The findings of the drug sensitivity analysis indicated that CCT5 expression, which is linked to the efficacy of various drugs, may play a crucial role in the future development of targeted immunotherapies for cancers characterized by CCT5 expression. CCT5-related gene enrichment analysis offers potential mechanisms by which CCT5 regulates the cell cycle pathway and various cellular functions in cancer. Further experimental and clinical studies are warranted to investigate CCT5’s practical application in cancer therapy and prognosis prediction.

## Electronic supplementary material

Below is the link to the electronic supplementary material.

Supplementary Material 1

Supplementary Material 2

Supplementary Material 3

Supplementary Material 4

Supplementary Material 5

Supplementary Material 6

## Data Availability

All data generated or analyzed during this study are included in this article and its supplementary information files. These data are also available in the following databases: GEPIA2 (http://gepia2.cancer-pku.cn/#index), SMART (http://www.bioinfo-zs.com/smartapp/), TIMER2.0 (http://timer.cistrome.org/), UALCAN (https://ualcan.path.uab.edu/), cBioportal (https://www.cbioportal.org/), Enrichr (https://maayanlab.cloud/Enrichr/), and the Ensembl Database (https://www.ensembl.org/index.html). GSCA (Gene Set Cancer Analysis) database (https://guolab.wchscu.cn/GSCA/#/), Tumor Immune Single-cell Hub 2 (TISCH2) (http://tisch.comp-genomics.org/home/), STRING database (https://string-db.org/), InteractiVenn - Interactive Venn Diagrams (https://www.interactivenn.net/), and TCGAplot (v8.0.0) R package (https://github.com/tjhwangxiong/TCGAplot).
