# Supplementary material for: Pan-cancer analysis reveals immunological and prognostic significance of CCT5 in human tumors
Source: Sci Rep. 2025 Apr 24;15:14405. doi: 10.1038/s41598-025-88339-z (PMC12022336; doi:10.1038/s41598-025-88339-z)
Supplement: Supplementary file 2 — Supplementary Material 2 [file 41598_2025_88339_MOESM2_ESM.pdf]

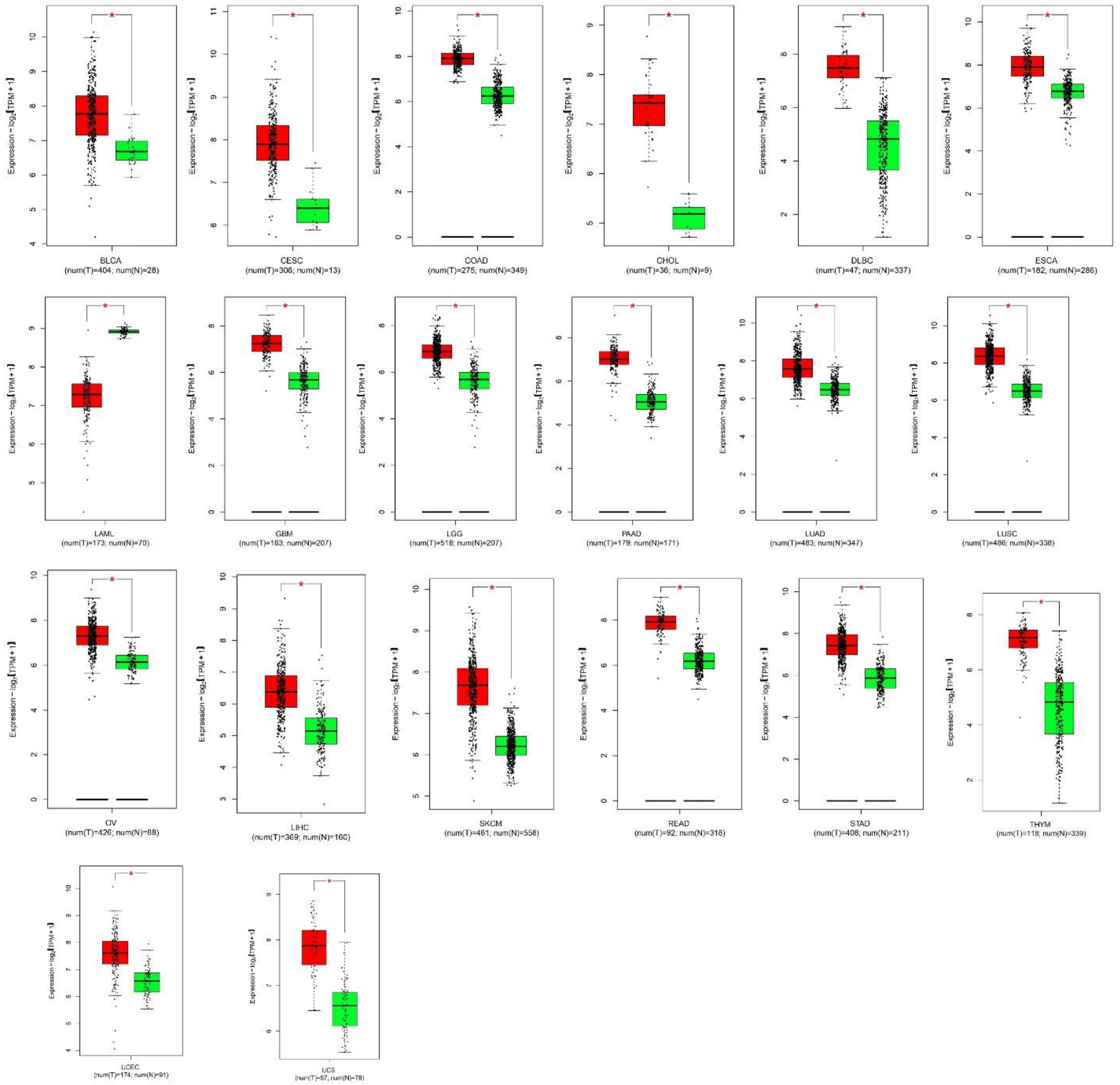

Supplementary Figure 1: Differential expression analysis conducted by GEPIA2 (http://gepia2.cancer-pku.cn/). Red box plots indicate tumors and greens indicate correspondent normal tissues. Significant over-expression of CCT5 in BLCA, CESC, COAD, CHOL, DLBC, ESCA, GBM, LGG, PAAD, LUAD, LUSC, OV, LIHC, SKCM, READ, STAD, THYM, UCEC and, UCS. Significant under-expression found only in LAML.

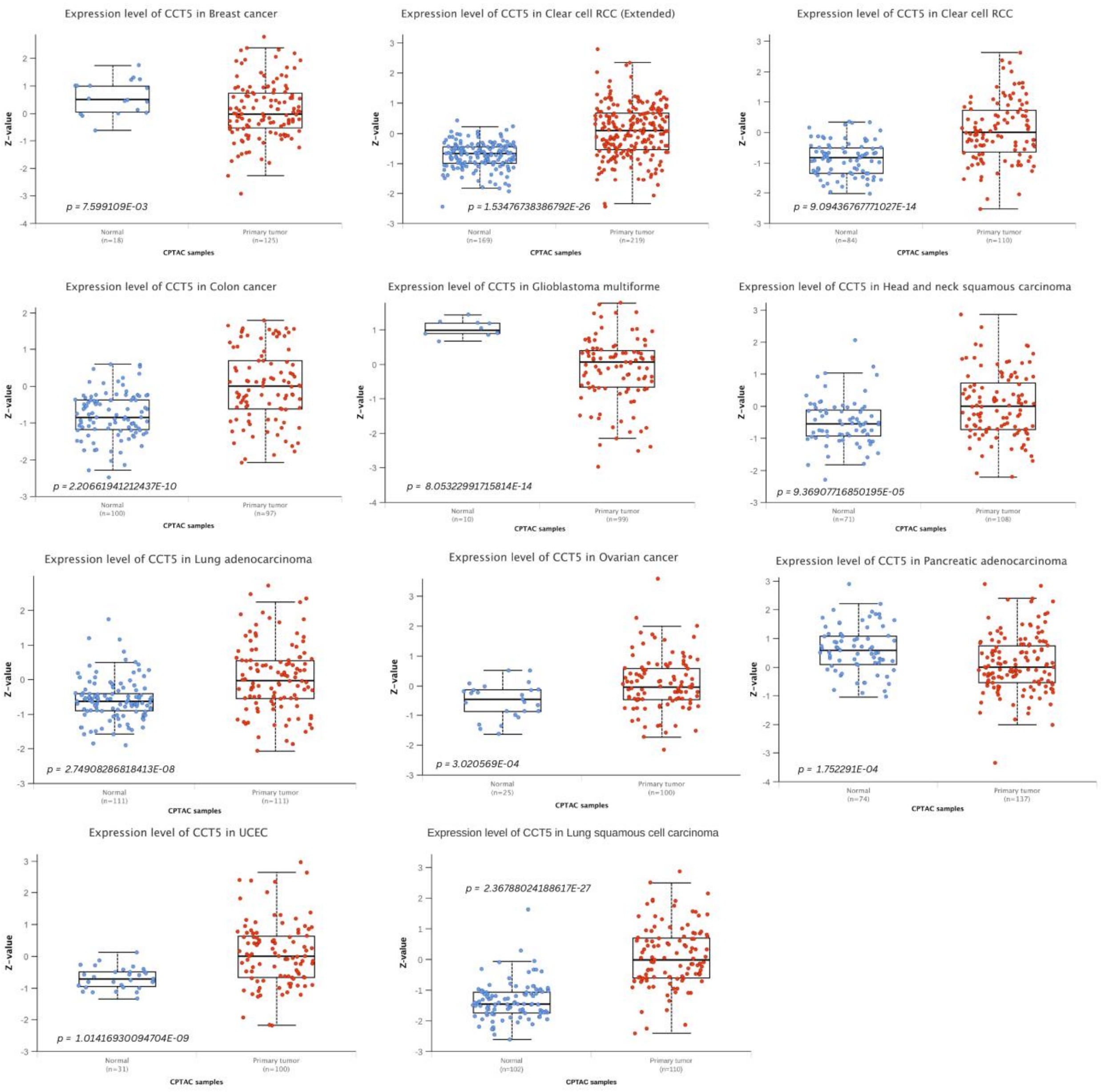

Supplementary Figure 2: Proteomic expression analysis of breast cancer, clear cell RCC, clear cell RCC (extended), Colon cancer, glioblastoma multiforme, head and neck squamous carcinoma, lung adenocarcinoma, lung squamous cell carcinoma, ovarian cancer, pancreatic adenocarcinoma, and UCEC was performed using the UALCAN (https://ualcan.path.uab.edu/) database ($p < 0.05$).

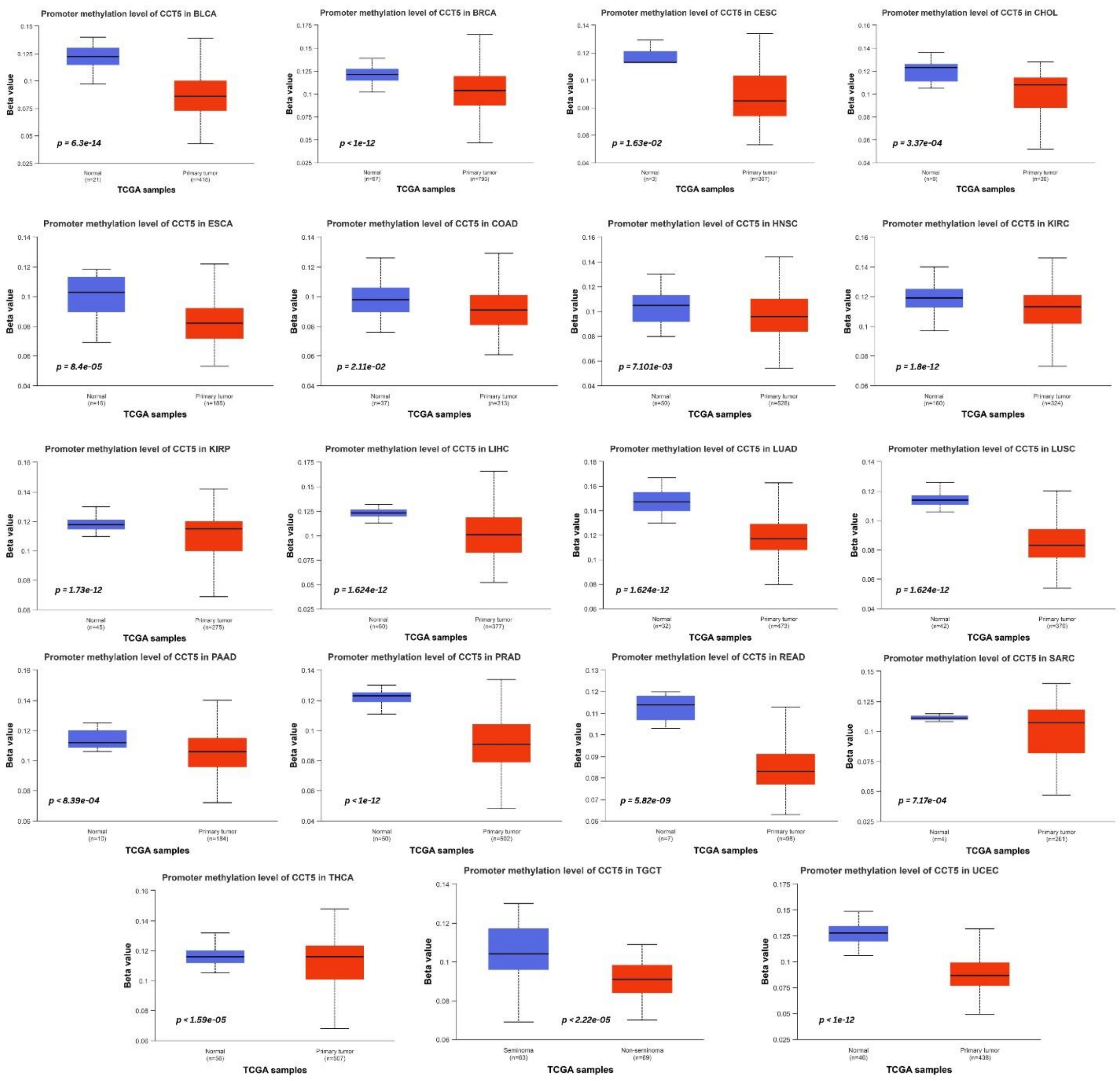

Supplementary Figure 3: DNA promoter methylation analysis of CCT5 with different tumor types analyzed by UALCAN (https://ualcan.path.uab.edu/). Significant promoter methylation level downregulated in correspondent tumor types including BLCA, BRCA, CESC, CHOL, COAD, ESCA, HNSC, KIRP, KIRC, LIHC, LUAD, LUSC, PAAD, PRAD, READ, SARC, TGCT, THCA and UCEC.

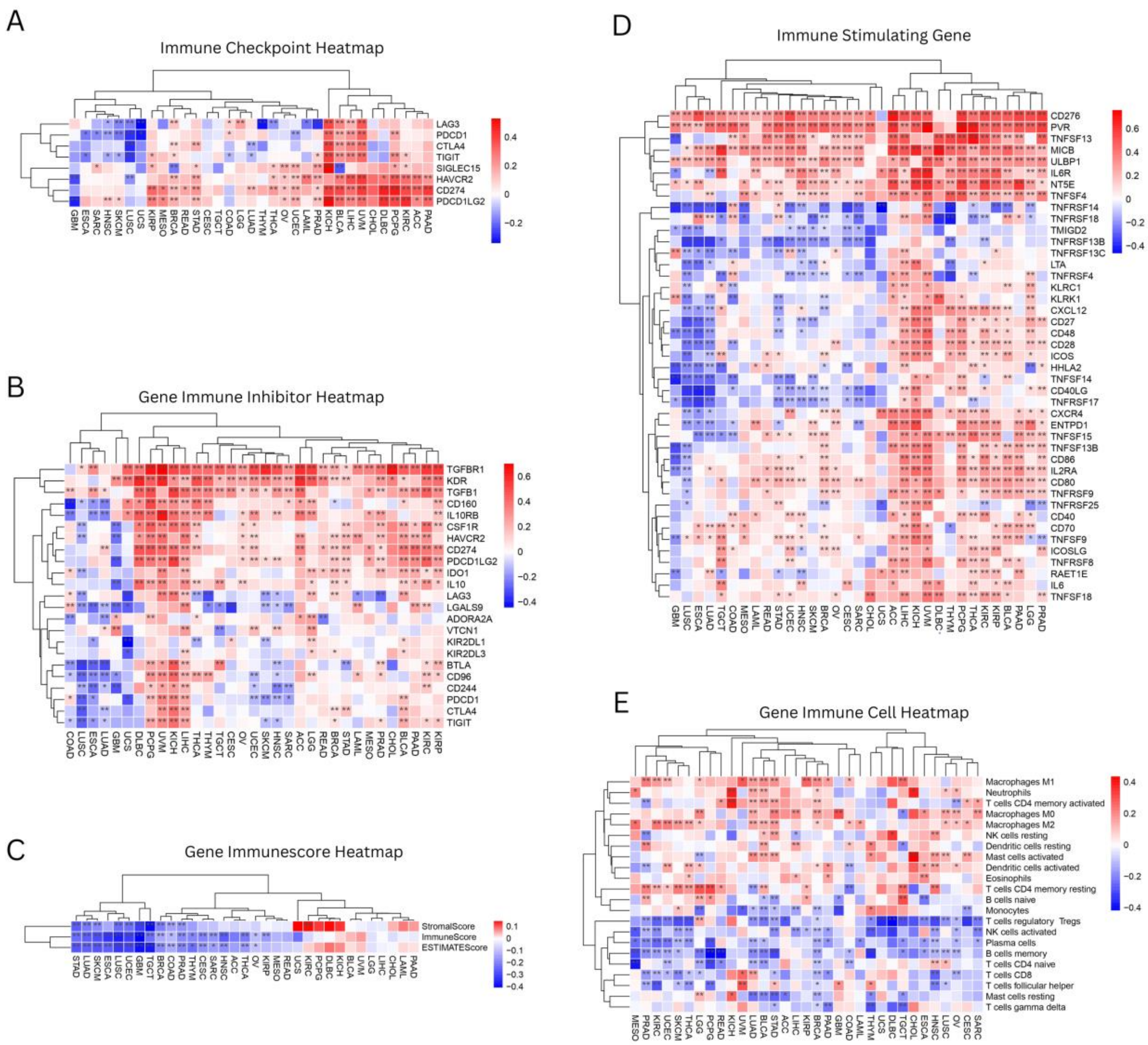

Supplementary Figure 4: Pan-cancer correlation with CCT5 and immune microenvironment. (A) Immune checkpoint genes heatmap, (B) Immune inhibitory genes heatmap, (C) Immune score heatmap, (D) Immune stimulating genes heatmap, (E) Immune cell, association with CCT5 expression. (*$p < 0.05$, **$p < 0.01$). The heatmap was generated utilizing the previously published TCGAplot (v8.0.0) R package (https://github.com/tjhwangxiong/TCGAplot)

Table 1: Gene Ontology Biological Process 2023

Index	Name	*p-value*	Adjusted *p-value*	Odds ratio	Combined score
1	Protein Stabilization (GO:0050821)	8.463e-17	5.583e-14	16.22	600.11
2	Chaperone-Mediated Protein Complex Assembly (GO:0051131)	1.275e-16	5.583e-14	129.76	4748.99
3	Mitotic Sister Chromatid Segregation (GO:0000070)	1.763e-16	5.583e-14	25.02	907.49
4	Positive Regulation Of DNA Biosynthetic Process (GO:2000573)	1.506e-15	3.576e-13	36.40	1242.15
5	Positive Regulation Of Telomerase RNA Localization To Cajal Body (GO:1904874)	2.269e-14	4.311e-12	187.66	5895.69
6	Positive Regulation Of Establishment Of Protein Localization To Telomere (GO:1904851)	3.928e-14	6.219e-12	489.24	15101.82
7	Regulation Of Establishment Of Protein Localization To Telomere (GO:0070203)	1.301e-13	1.373e-11	326.14	9676.73
8	Regulation Of Protein Localization To Cajal Body (GO:1904869)	1.301e-13	1.373e-11	326.14	9676.73
9	Positive Regulation Of Protein Localization To Cajal Body (GO:1904871)	1.301e-13	1.373e-11	326.14	9676.73
10	Regulation Of Telomerase RNA Localization To Cajal Body (GO:1904872)	1.802e-13	1.712e-11	125.09	3670.62

Table 2: Gene Ontology Cellular Component 2023

Index	Name	*p-value*	Adjusted *p-value*	Odds ratio	Combined score
1	Microtubule Cytoskeleton (GO:0015630)	6.663e-18	6.796e-16	12.42	491.36

2	Microtubule (GO:0005874)	1.519e-16	7.745e-15	17.33	631.28
3	Spindle (GO:0005819)	3.383e-13	8.819e-12	13.12	376.67
4	Intracellular Non-Membrane-Bounded Organelle (GO:0043232)	3.459e-13	8.819e-12	5.14	147.42
5	Nucleus (GO:0005634)	9.385e-11	1.915e-9	3.01	69.57
6	Polymeric Cytoskeletal Fiber (GO:0099513)	1.457e-10	2.478e-9	9.47	214.50
7	Mitotic Spindle (GO:0072686)	7.693e-10	1.121e-8	13.19	276.70
8	Intracellular Membrane-Bounded Organelle (GO:0043231)	8.154e-9	1.040e-7	2.63	48.98
9	Spindle Microtubule (GO:0005876)	3.980e-8	4.510e-7	18.71	318.89
10	Nuclear Lumen (GO:0031981)	0.000005635	0.00005748	3.67	44.31

Table 3: Gene Ontology Molecular Function 2023

Index	Name	*p-value*	Adjusted *p-value*	Odds ratio	Combined score
1	RNA Binding (GO:0003723)	1.478e-7	0.00002454	3.37	53.03
2	Microtubule Binding (GO:0008017)	0.00001259	0.001045	6.16	69.55
3	Amyloid-Beta Binding (GO:0001540)	0.00002746	0.001265	11.37	119.39
4	Tubulin Binding (GO:0015631)	0.00003047	0.001265	5.01	52.08
5	Protein Serine/Threonine Phosphatase Activity (GO:0004722)	0.00005529	0.001836	13.75	134.80
6	Hsp90 Protein Binding (GO:0051879)	0.0001294	0.003581	17.64	157.90
7	Kinase Binding (GO:0019900)	0.0001754	0.004122	3.79	32.81

8	Tau Protein Binding (GO:0048156)	0.0001987	0.004122	15.62	133.13
9	Protein Serine/Threonine Kinase Activity (GO:0004674)	0.0002507	0.004623	4.23	35.07
10	Cyclin-Dependent Protein Serine/Threonine Kinase Regulator Activity (GO:0016538)	0.0003785	0.006284	13.01	102.51

Table 4: Reactome 2022

Index	Name	*p-value*	Adjusted *p-value*	Odds ratio	Combined score
1	Cell Cycle R-HSA-1640170	2.982e-32	1.876e-29	14.13	1026.05
2	Cell Cycle, Mitotic R-HSA-69278	1.767e-30	5.556e-28	15.26	1045.12
3	Prefoldin Mediated Transfer Of Substrate To CCT/TriC R-HSA-389957	1.107e-27	2.321e-25	198.89	12344.55
4	Cooperation Of Prefoldin And TriC/CCT In Actin And Tubulin Folding R-HSA-389958	4.116e-26	6.472e-24	140.36	8204.13
5	Chaperonin-mediated Protein Folding R-HSA-390466	5.286e-26	6.650e-24	47.04	2737.60
6	Protein Folding R-HSA-391251	2.346e-25	2.459e-23	43.26	2453.36
7	M Phase R-HSA-68886	1.100e-24	9.880e-23	15.33	845.60
8	Mitotic Anaphase R-HSA-68882	5.092e-22	3.963e-20	19.13	938.06
9	Mitotic Metaphase And Anaphase R-HSA-2555396	5.670e-22	3.963e-20	19.04	931.46
10	Resolution Of Sister Chromatid Cohesion R-HSA-2500257	2.922e-18	1.838e-16	28.60	1154.57

Table 5: WikiPathway 2024 Human

Index	Name	*p-value*	Adjusted *p-value*	Odds ratio	Combined score
1	Cellular Proteostasis WP4918	1.545e-13	2.672e-11	119106.00	3513489.47
2	16P11 2 Proximal Deletion Syndrome WP4949	1.856e-10	1.605e-8	22.24	498.40
3	Cell Cycle WP179	1.566e-9	9.031e-8	14.44	292.71
4	Retinoblastoma Gene In Cancer WP2446	1.734e-8	7.502e-7	16.30	291.22
5	Cohesin Complex Cornelia De Lange Syndrome WP5117	0.000005015	0.0001735	23.73	289.62
6	ATM Signaling In Development And Disease WP3878	0.00002558	0.0007375	16.38	173.16
7	Gastric Cancer Network 1 WP2361	0.00004544	0.001123	23.78	237.80
8	Gastric Cancer Network 2 WP2363	0.00007960	0.001721	20.25	191.17
9	Regulation Sister Chromatid Sep At Meta-Anaphase Transition WP4240	0.0001389	0.002584	37.06	329.17
10	miRNA Regulation Of DNA Damage Response WP1530	0.0001643	0.002584	10.74	93.54

Table 6: KEGG Human 2021

Index	Name	*p-value*	Adjusted *p-value*	Odds ratio	Combined score
1	Cell cycle	1.455e-10	1.761e-8	15.44	349.66
2	Oocyte meiosis	4.484e-8	0.000002390	11.93	201.84

3	Progesterone-mediated oocyte maturation	5.926e-8	0.000002390	13.96	232.30
4	Amyotrophic lateral sclerosis	0.0004104	0.01202	3.96	30.90
5	RNA transport	0.0004966	0.01202	5.42	41.21
6	Cellular senescence	0.001123	0.02234	5.51	37.43
7	Ribosome biogenesis in eukaryotes	0.001292	0.02234	6.66	44.28
8	AMPK signaling pathway	0.002058	0.03112	5.96	36.86
9	Dopaminergic synapse	0.003111	0.04182	5.39	31.13
10	Apoptosis	0.004248	0.04876	5.00	27.29
