# Supplementary figures and images for: Pan-cancer analysis reveals immunological and prognostic significance of CCT5 in human tumors
Source: Sci Rep. 2025 Apr 24;15:14405. doi: 10.1038/s41598-025-88339-z (PMC12022336; doi:10.1038/s41598-025-88339-z)

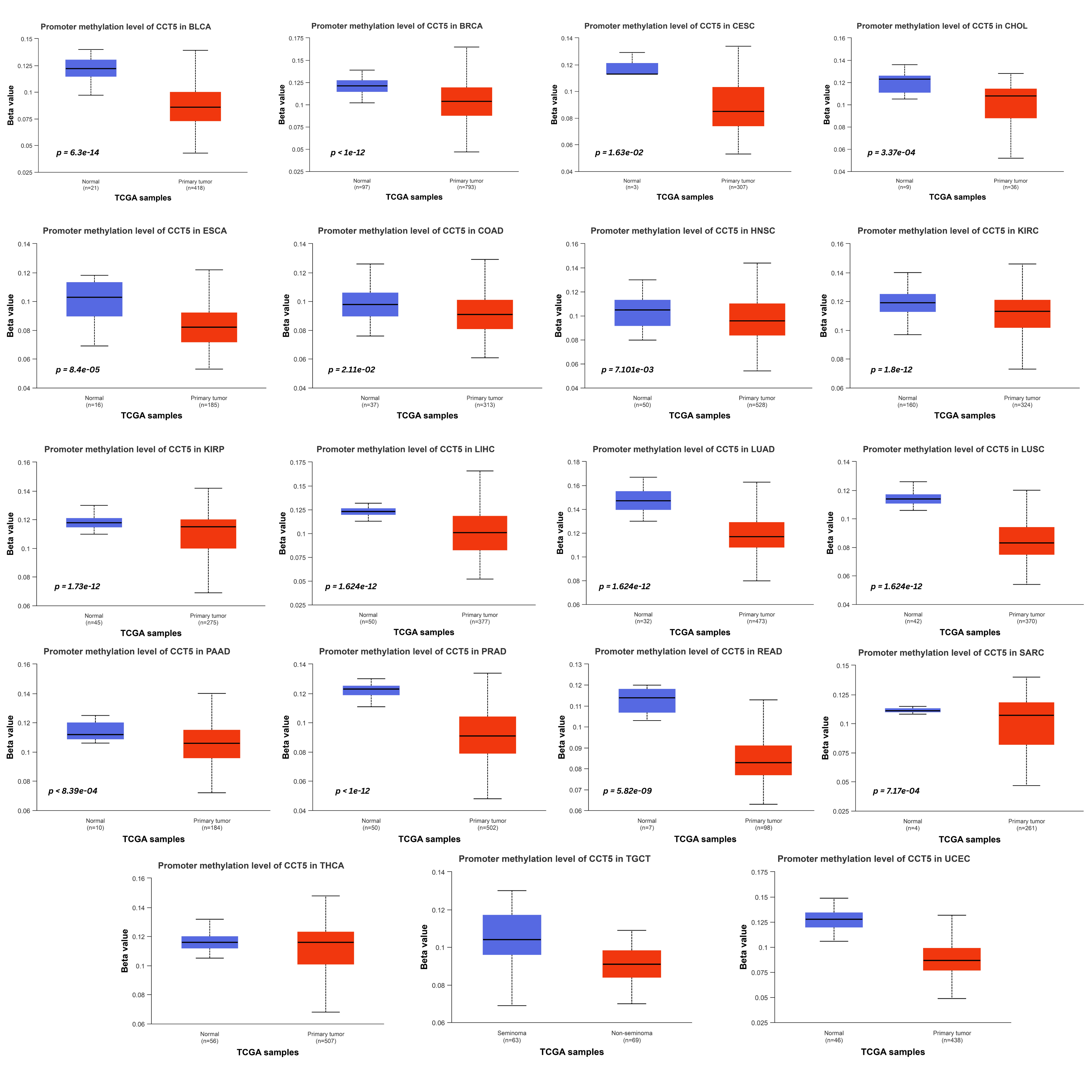

Supplement: Supplementary file 3 — Supplementary Material 3 [file 41598_2025_88339_MOESM3_ESM.png]

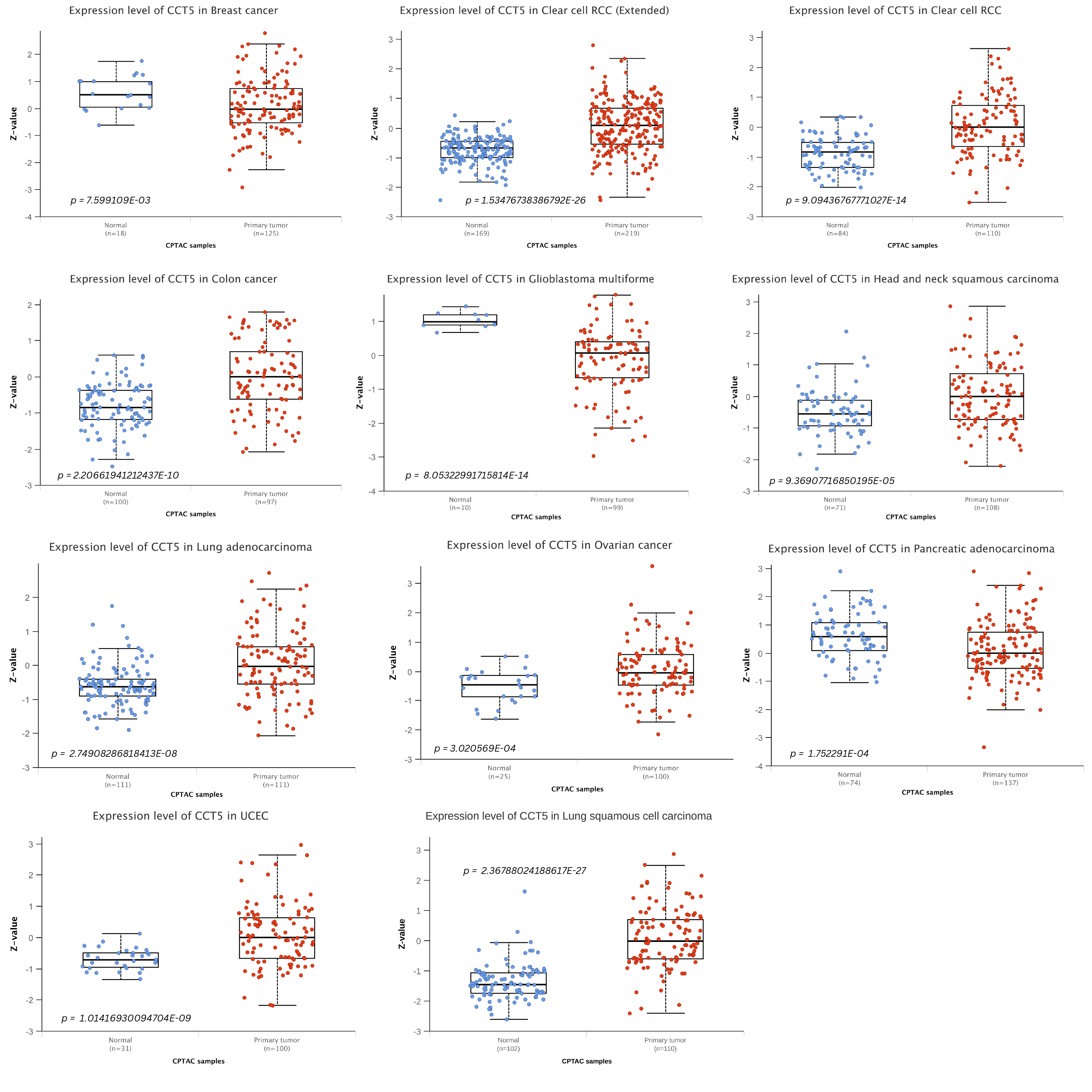

Supplement: Supplementary file 4 — Supplementary Material 4 [file 41598_2025_88339_MOESM4_ESM.jpg]

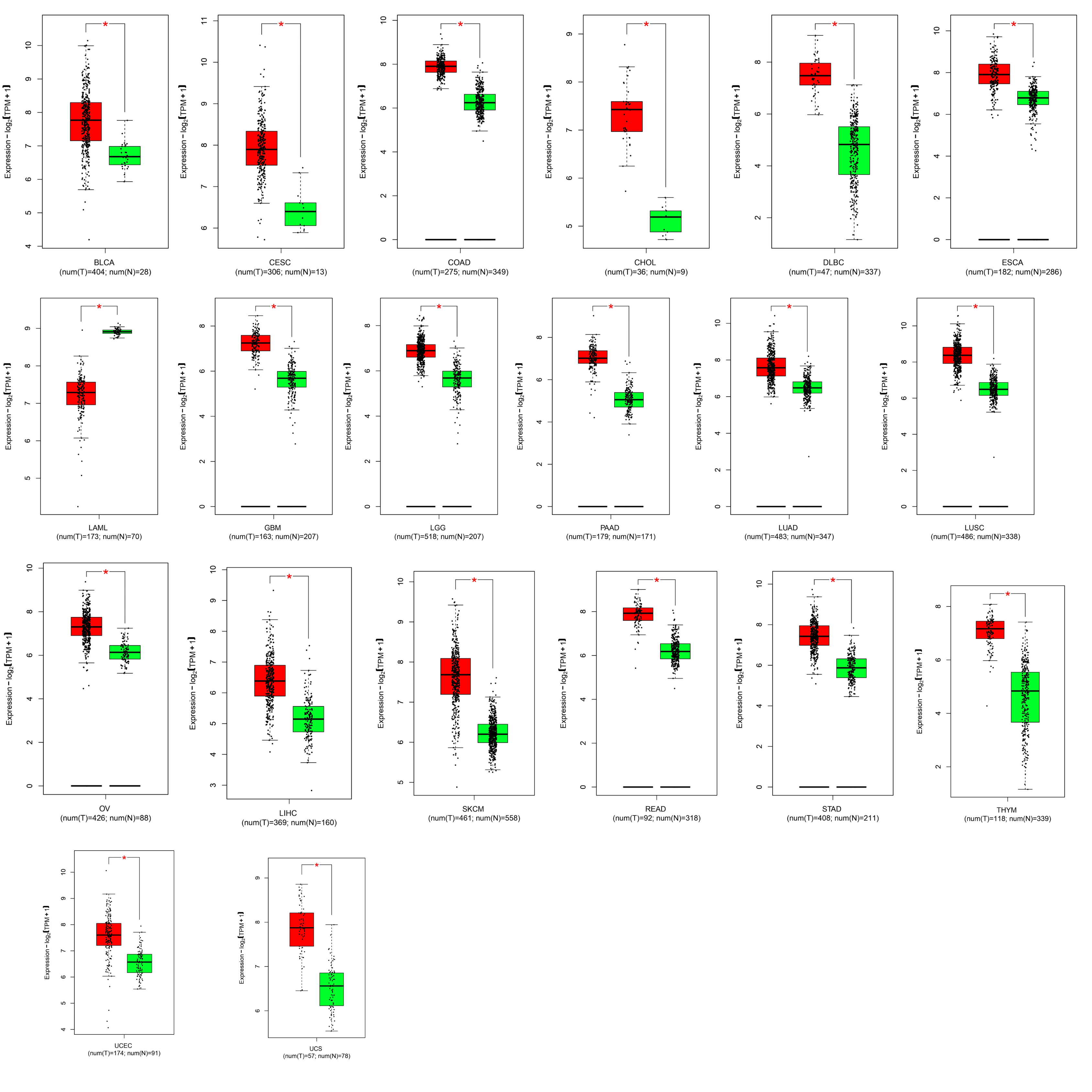

Supplement: Supplementary file 5 — Supplementary Material 5 [file 41598_2025_88339_MOESM5_ESM.png]

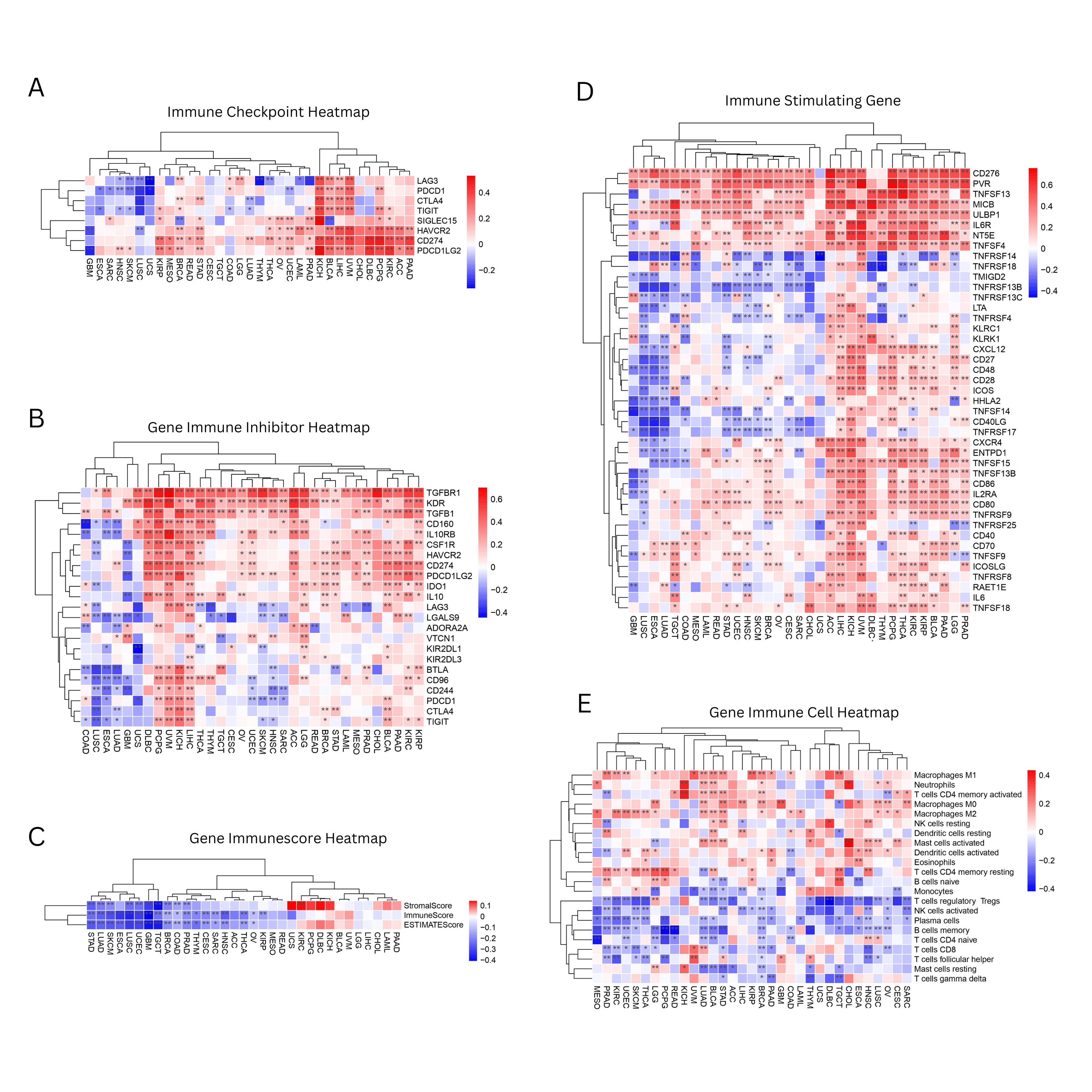

Supplement: Supplementary file 6 — Supplementary Material 6 [file 41598_2025_88339_MOESM6_ESM.png]
